# Supplementary material for: Application of synthesized metal-trimesic acid frameworks for the remediation of a multi-metal polluted soil and investigation of quinoa responses
Source: PLoS One. 2024 Sep 6;19(9):e0310054. doi: 10.1371/journal.pone.0310054 (PMC11379216; doi:10.1371/journal.pone.0310054)
Supplement: S1 Table — (DOCX) [file pone.0310054.s001.docx]

| **Treatments** | **SPSS code** (VAR00001) | **Replications** | **Metal uptake (µg pot^-1^)** | | | |
| --- | --- | --- | --- | --- | --- | --- |
|  |  |  | **Zn** | **Ni** | **Pb** | **Cd** |
| Control | 1.00 | Rep 1 | 188.34 | 148.44 | 189.59 | 175.17 |
|  | 1.00 | Rep 2 | 172.15 | 157.25 | 192.46 | 166.11 |
|  | 1.00 | Rep 3 | 183.8 | 145.3 | 182.25 | 158.19 |
| Zn-BTC (1%) | 2.00 | Rep 1 | 31.43 | 11.01 | 35.26 | 16.95 |
|  | 2.00 | Rep 2 | 28.56 | 14.44 | 29.14 | 19.11 |
|  | 2.00 | Rep 3 | 30.85 | 9.26 | 32.77 | 22.14 |
| Zn-BTC (0.5%) | 3.00 | Rep 1 | 37.35 | 21.55 | 57.56 | 46.11 |
|  | 3.00 | Rep 2 | 40.14 | 21.93 | 51.19 | 51.66 |
|  | 3.00 | Rep 3 | 40.83 | 26.6 | 54.15 | 54 |
| Cu-BTC (1%) | 4.00 | Rep 1 | 8.35 | 8.14 | 3.11 | 6.14 |
|  | 4.00 | Rep 2 | 9.14 | 8.91 | 5.65 | 5.88 |
|  | 4.00 | Rep 3 | 9.48 | 9.86 | 5.85 | 7.36 |
| Cu-BTC (0.5%) | 5.00 | Rep 1 | 13.75 | 13.88 | 7.08 | 8.11 |
|  | 5.00 | Rep 2 | 13.01 | 12.21 | 10.14 | 11.14 |
|  | 5.00 | Rep 3 | 15.81 | 18.73 | 6.36 | 10.54 |
| Fe-BTC (1%) | 6.00 | Rep 1 | 69.45 | 66.18 | 90.48 | 70.11 |
|  | 6.00 | Rep 2 | 64.77 | 65.34 | 78.11 | 60.88 |
|  | 6.00 | Rep 3 | 60.81 | 63.66 | 82.15 | 68.63 |
| Fe-BTC (0.5%) | 7.00 | Rep 1 | 61.25 | 54.58 | 79.01 | 59.17 |
|  | 7.00 | Rep 2 | 64.31 | 68.15 | 70.19 | 63.34 |
|  | 7.00 | Rep 3 | 65.9 | 61.08 | 76.91 | 65.08 |

**S1 Table. Data related to the uptake of HMs by shoot.**

| **Zn-Shoot** | | | | | | |
| --- | --- | --- | --- | --- | --- | --- |
| Duncan^a^ | | | | | | |
| VAR00001 | N | Subset for alpha = 0.05 | | | | |
|  |  | 1 | 2 | 3 | 4 | 5 |
| 4.00 | 3 | 8.9900 |  |  |  |  |
| 5.00 | 3 | 14.1900 |  |  |  |  |
| 2.00 | 3 |  | 30.2800 |  |  |  |
| 3.00 | 3 |  |  | 39.4400 |  |  |
| 7.00 | 3 |  |  |  | 63.8200 |  |
| 6.00 | 3 |  |  |  | 65.0100 |  |
| 1.00 | 3 |  |  |  |  | 181.4300 |
| Sig. |  | .118 | 1.000 | 1.000 | .709 | 1.000 |
| Means for groups in homogeneous subsets are displayed. | | | | | | |
| a. Uses Harmonic Mean Sample Size = 3,000. | | | | | | |

| **Ni-Shoot** | | | | | |
| --- | --- | --- | --- | --- | --- |
| Duncan^a^ | | | | | |
| VAR00001 | N | Subset for alpha = 0.05 | | | |
|  |  | 1 | 2 | 3 | 4 |
| 4.00 | 3 | 8.9700 |  |  |  |
| 2.00 | 3 | 11.5700 |  |  |  |
| 5.00 | 3 | 14.9400 |  |  |  |
| 3.00 | 3 |  | 23.3600 |  |  |
| 7.00 | 3 |  |  | 61.2700 |  |
| 6.00 | 3 |  |  | 65.0600 |  |
| 1.00 | 3 |  |  |  | 150.3300 |
| Sig. |  | .105 | 1.000 | .268 | 1.000 |
| Means for groups in homogeneous subsets are displayed. | | | | | |
| a. Uses Harmonic Mean Sample Size = 3,000. | | | | | |

| **Pb-Shoot** | | | | | | | |
| --- | --- | --- | --- | --- | --- | --- | --- |
| Duncan^a^ | | | | | | | |
| VAR00001 | N | Subset for alpha = 0.05 | | | | | |
|  |  | 1 | 2 | 3 | 4 | 5 | 6 |
| 4.00 | 3 | 4.8700 |  |  |  |  |  |
| 5.00 | 3 | 7.8600 |  |  |  |  |  |
| 2.00 | 3 |  | 32.3900 |  |  |  |  |
| 3.00 | 3 |  |  | 54.3000 |  |  |  |
| 7.00 | 3 |  |  |  | 75.3700 |  |  |
| 6.00 | 3 |  |  |  |  | 83.5800 |  |
| 1.00 | 3 |  |  |  |  |  | 188.1000 |
| Sig. |  | .381 | 1.000 | 1.000 | 1.000 | 1.000 | 1.000 |
| Means for groups in homogeneous subsets are displayed. | | | | | | | |
| a. Uses Harmonic Mean Sample Size = 3,000. | | | | | | | |

| **Cd-Shoot** | | | | | | |
| --- | --- | --- | --- | --- | --- | --- |
| Duncan^a^ | | | | | | |
| VAR00001 | N | Subset for alpha = 0.05 | | | | |
|  |  | 1 | 2 | 3 | 4 | 5 |
| 4.00 | 3 | 6.4600 |  |  |  |  |
| 5.00 | 3 | 9.9300 |  |  |  |  |
| 2.00 | 3 |  | 19.4000 |  |  |  |
| 3.00 | 3 |  |  | 50.5900 |  |  |
| 7.00 | 3 |  |  |  | 62.5300 |  |
| 6.00 | 3 |  |  |  | 66.5400 |  |
| 1.00 | 3 |  |  |  |  | 166.4900 |
| Sig. |  | .345 | 1.000 | 1.000 | .278 | 1.000 |
| Means for groups in homogeneous subsets are displayed. | | | | | | |
| a. Uses Harmonic Mean Sample Size = 3,000. | | | | | | |
