# Supplementary material for: Application of synthesized metal-trimesic acid frameworks for the remediation of a multi-metal polluted soil and investigation of quinoa responses
Source: PLoS One. 2024 Sep 6;19(9):e0310054. doi: 10.1371/journal.pone.0310054 (PMC11379216; doi:10.1371/journal.pone.0310054)
Supplement: S2 Table — (DOCX) [file pone.0310054.s002.docx]

| **Treatments** | **SPSS code** (VAR00001) | **Replications** | **Metal uptake (µg pot^-1^)** | | | |
| --- | --- | --- | --- | --- | --- | --- |
|  |  |  | **Zn** | **Ni** | **Pb** | **Cd** |
| Control | 1.00 | Rep 1 | 114.46 | 153.23 | 32.96 | 71.48 |
|  | 1.00 | Rep 2 | 124.16 | 134.36 | 35.26 | 70.28 |
|  | 1.00 | Rep 3 | 120.45 | 147.23 | 36 | 74.39 |
| Zn-BTC (1%) | 2.00 | Rep 1 | 3.26 | 2.11 | 1.41 | 3.98 |
|  | 2.00 | Rep 2 | 3.53 | 2.64 | 1.12 | 4.75 |
|  | 2.00 | Rep 3 | 3.29 | 2.9 | 1.22 | 4.47 |
| Zn-BTC (0.5%) | 3.00 | Rep 1 | 9.21 | 86.78 | 32.84 | 37.96 |
|  | 3.00 | Rep 2 | 9.46 | 79.96 | 30.26 | 36.14 |
|  | 3.00 | Rep 3 | 13.43 | 92.13 | 28.64 | 38.76 |
| Cu-BTC (1%) | 4.00 | Rep 1 | 1.53 | 4.78 | 0.39 | 0.94 |
|  | 4.00 | Rep 2 | 1.75 | 4.58 | 0.37 | 1.18 |
|  | 4.00 | Rep 3 | 2.24 | 5.55 | 0.41 | 0.97 |
| Cu-BTC (0.5%) | 5.00 | Rep 1 | 2.97 | 9.98 | 3.98 | 3.12 |
|  | 5.00 | Rep 2 | 3.54 | 7.75 | 2.98 | 4.02 |
|  | 5.00 | Rep 3 | 3.9 | 8.46 | 3.33 | 3.18 |
| Fe-BTC (1%) | 6.00 | Rep 1 | 24.84 | 58.24 | 11.14 | 23.68 |
|  | 6.00 | Rep 2 | 22.26 | 52.78 | 12.81 | 24.47 |
|  | 6.00 | Rep 3 | 19.29 | 55.36 | 13.4 | 28.14 |
| Fe-BTC (0.5%) | 7.00 | Rep 1 | 15.98 | 48.44 | 8.24 | 25.17 |
|  | 7.00 | Rep 2 | 15.23 | 53.11 | 7.08 | 24.19 |
|  | 7.00 | Rep 3 | 13.1 | 52.98 | 7.93 | 23 |

**S2 Table. Data related to the uptake of HMs by root.**

| **Zn-Root** | | | | | |
| --- | --- | --- | --- | --- | --- |
| Duncan^a^ | | | | | |
| VAR00001 | N | Subset for alpha = 0.05 | | | |
|  |  | 1 | 2 | 3 | 4 |
| 4.00 | 3 | 1.8400 |  |  |  |
| 2.00 | 3 | 3.3600 |  |  |  |
| 5.00 | 3 | 3.4700 |  |  |  |
| 3.00 | 3 |  | 10.7000 |  |  |
| 7.00 | 3 |  | 14.7700 |  |  |
| 6.00 | 3 |  |  | 22.1300 |  |
| 1.00 | 3 |  |  |  | 119.6900 |
| Sig. |  | .441 | .056 | 1.000 | 1.000 |
| Means for groups in homogeneous subsets are displayed. | | | | | |
| a. Uses Harmonic Mean Sample Size = 3,000. | | | | | |

| **Ni-Root** | | | | | |
| --- | --- | --- | --- | --- | --- |
| Duncan^a^ | | | | | |
| VAR00001 | N | Subset for alpha = 0.05 | | | |
|  |  | 1 | 2 | 3 | 4 |
| 2.00 | 3 | 2.5500 |  |  |  |
| 4.00 | 3 | 4.9700 |  |  |  |
| 5.00 | 3 | 8.7300 |  |  |  |
| 7.00 | 3 |  | 51.5100 |  |  |
| 6.00 | 3 |  | 55.4600 |  |  |
| 3.00 | 3 |  |  | 86.2900 |  |
| 1.00 | 3 |  |  |  | 144.9400 |
| Sig. |  | .138 | .308 | 1.000 | 1.000 |
| Means for groups in homogeneous subsets are displayed. | | | | | |
| a. Uses Harmonic Mean Sample Size = 3,000. | | | | | |

| **Pb-Root** | | | | | | | |
| --- | --- | --- | --- | --- | --- | --- | --- |
| Duncan^a^ | | | | | | | |
| VAR00001 | N | Subset for alpha = 0.05 | | | | | |
|  |  | 1 | 2 | 3 | 4 | 5 | 6 |
| 4.00 | 3 | .3900 |  |  |  |  |  |
| 2.00 | 3 | 1.2500 |  |  |  |  |  |
| 5.00 | 3 |  | 3.4300 |  |  |  |  |
| 7.00 | 3 |  |  | 7.7500 |  |  |  |
| 6.00 | 3 |  |  |  | 12.4500 |  |  |
| 3.00 | 3 |  |  |  |  | 30.5800 |  |
| 1.00 | 3 |  |  |  |  |  | 34.7400 |
| Sig. |  | .369 | 1.000 | 1.000 | 1.000 | 1.000 | 1.000 |
| Means for groups in homogeneous subsets are displayed. | | | | | | | |
| a. Uses Harmonic Mean Sample Size = 3,000. | | | | | | | |

| **Cd-Root** | | | | | | |
| --- | --- | --- | --- | --- | --- | --- |
| Duncan^a^ | | | | | | |
| VAR00001 | N | Subset for alpha = 0.05 | | | | |
|  |  | 1 | 2 | 3 | 4 | 5 |
| 4.00 | 3 | 1.0300 |  |  |  |  |
| 5.00 | 3 | 3.4400 | 3.4400 |  |  |  |
| 2.00 | 3 |  | 4.4000 |  |  |  |
| 7.00 | 3 |  |  | 24.1200 |  |  |
| 6.00 | 3 |  |  | 25.4300 |  |  |
| 3.00 | 3 |  |  |  | 37.6200 |  |
| 1.00 | 3 |  |  |  |  | 72.0500 |
| Sig. |  | .052 | .412 | .268 | 1.000 | 1.000 |
| Means for groups in homogeneous subsets are displayed. | | | | | | |
| a. Uses Harmonic Mean Sample Size = 3,000. | | | | | | |
