# Supplementary material for: Application of synthesized metal-trimesic acid frameworks for the remediation of a multi-metal polluted soil and investigation of quinoa responses
Source: PLoS One. 2024 Sep 6;19(9):e0310054. doi: 10.1371/journal.pone.0310054 (PMC11379216; doi:10.1371/journal.pone.0310054)
Supplement: S3 Table — (DOCX) [file pone.0310054.s003.docx]

| **Treatments** | **SPSS code** (VAR00001) | **Replications** | **Metal concentration (mg kg^-1^)** | | | |
| --- | --- | --- | --- | --- | --- | --- |
|  |  |  | **Zn** | **Ni** | **Pb** | **Cd** |
| Control | 1.00 | Rep 1 | 54.24 | 42.42 | 60.12 | 61.97 |
|  | 1.00 | Rep 2 | 55.95 | 43.55 | 59.46 | 60 |
|  | 1.00 | Rep 3 | 60.21 | 42.22 | 62.82 | 66.01 |
| Zn-BTC (1%) | 2.00 | Rep 1 | 50.28 | 32.81 | 46.12 | 56.11 |
|  | 2.00 | Rep 2 | 51.48 | 32.41 | 48.93 | 48.86 |
|  | 2.00 | Rep 3 | 55.8 | 34.11 | 50 | 52.59 |
| Zn-BTC (0.5%) | 3.00 | Rep 1 | 47.51 | 39.41 | 55.89 | 60.88 |
|  | 3.00 | Rep 2 | 49.21 | 40.16 | 54.75 | 55.46 |
|  | 3.00 | Rep 3 | 49.95 | 40.25 | 57.3 | 57.66 |
| Cu-BTC (1%) | 4.00 | Rep 1 | 16 | 16.88 | 10.26 | 14.85 |
|  | 4.00 | Rep 2 | 15.88 | 17.9 | 12.65 | 11.38 |
|  | 4.00 | Rep 3 | 17.05 | 17.33 | 12.67 | 12.87 |
| Cu-BTC (0.5%) | 5.00 | Rep 1 | 27.55 | 23.12 | 19.56 | 20.14 |
|  | 5.00 | Rep 2 | 29.21 | 25.14 | 17.88 | 24.89 |
|  | 5.00 | Rep 3 | 28.56 | 24.4 | 21.36 | 22.95 |
| Fe-BTC (1%) | 6.00 | Rep 1 | 43.55 | 35.65 | 45.13 | 59 |
|  | 6.00 | Rep 2 | 42.98 | 36.21 | 42.44 | 61.48 |
|  | 6.00 | Rep 3 | 44.51 | 34.97 | 44.51 | 63.12 |
| Fe-BTC (0.5%) | 7.00 | Rep 1 | 48.12 | 39.75 | 51.21 | 64.96 |
|  | 7.00 | Rep 2 | 47.22 | 39.15 | 47.84 | 60.11 |
|  | 7.00 | Rep 3 | 51.06 | 39.39 | 49.87 | 62.25 |

**S3 Table. Data related to HMs extracted by EDTA.**

| **Zn-EDTA** | | | | | | |
| --- | --- | --- | --- | --- | --- | --- |
| Duncan^a^ | | | | | | |
| VAR00001 | N | Subset for alpha = 0.05 | | | | |
|  |  | 1 | 2 | 3 | 4 | 5 |
| 4.00 | 3 | 16.3100 |  |  |  |  |
| 5.00 | 3 |  | 28.4400 |  |  |  |
| 6.00 | 3 |  |  | 43.6800 |  |  |
| 7.00 | 3 |  |  |  | 48.8000 |  |
| 3.00 | 3 |  |  |  | 48.8900 |  |
| 2.00 | 3 |  |  |  | 51.1867 |  |
| 1.00 | 3 |  |  |  |  | 56.8000 |
| Sig. |  | 1.000 | 1.000 | 1.000 | .160 | 1.000 |
| Means for groups in homogeneous subsets are displayed. | | | | | | |
| a. Uses Harmonic Mean Sample Size = 3,000. | | | | | | |

| **Ni-EDTA** | | | | | | | |
| --- | --- | --- | --- | --- | --- | --- | --- |
| Duncan^a^ | | | | | | | |
| VAR00001 | N | Subset for alpha = 0.05 | | | | | |
|  |  | 1 | 2 | 3 | 4 | 5 | 6 |
| 4.00 | 3 | 17.3700 |  |  |  |  |  |
| 5.00 | 3 |  | 24.2200 |  |  |  |  |
| 2.00 | 3 |  |  | 33.1100 |  |  |  |
| 6.00 | 3 |  |  |  | 35.6100 |  |  |
| 7.00 | 3 |  |  |  |  | 39.4300 |  |
| 3.00 | 3 |  |  |  |  | 39.9400 |  |
| 1.00 | 3 |  |  |  |  |  | 42.7967 |
| Sig. |  | 1.000 | 1.000 | 1.000 | 1.000 | .655 | 1.000 |
| Means for groups in homogeneous subsets are displayed. | | | | | | | |
| a. Uses Harmonic Mean Sample Size = 3,000. | | | | | | | |

| **Pb-EDTA** | | | | | | | |
| --- | --- | --- | --- | --- | --- | --- | --- |
| Duncan^a^ | | | | | | | |
| VAR00001 | N | Subset for alpha = 0.05 | | | | | |
|  |  | 1 | 2 | 3 | 4 | 5 | 6 |
| 4.00 | 3 | 11.8600 |  |  |  |  |  |
| 5.00 | 3 |  | 19.6000 |  |  |  |  |
| 6.00 | 3 |  |  | 44.0267 |  |  |  |
| 2.00 | 3 |  |  |  | 48.3500 |  |  |
| 7.00 | 3 |  |  |  | 49.6400 |  |  |
| 3.00 | 3 |  |  |  |  | 55.9800 |  |
| 1.00 | 3 |  |  |  |  |  | 60.8000 |
| Sig. |  | 1.000 | 1.000 | 1.000 | .349 | 1.000 | 1.000 |
| Means for groups in homogeneous subsets are displayed. | | | | | | | |
| a. Uses Harmonic Mean Sample Size = 3,000. | | | | | | | |

| **Cd-EDTA** | | | | | |
| --- | --- | --- | --- | --- | --- |
| Duncan^a^ | | | | | |
| VAR00001 | N | Subset for alpha = 0.05 | | | |
|  |  | 1 | 2 | 3 | 4 |
| 4.00 | 3 | 13.0333 |  |  |  |
| 5.00 | 3 |  | 22.6600 |  |  |
| 2.00 | 3 |  |  | 52.5200 |  |
| 3.00 | 3 |  |  |  | 58.0000 |
| 6.00 | 3 |  |  |  | 61.2000 |
| 7.00 | 3 |  |  |  | 62.4400 |
| 1.00 | 3 |  |  |  | 62.6600 |
| Sig. |  | 1.000 | 1.000 | 1.000 | .065 |
| Means for groups in homogeneous subsets are displayed. | | | | | |
| a. Uses Harmonic Mean Sample Size = 3,000. | | | | | |
