# Supplementary material for: Application of synthesized metal-trimesic acid frameworks for the remediation of a multi-metal polluted soil and investigation of quinoa responses
Source: PLoS One. 2024 Sep 6;19(9):e0310054. doi: 10.1371/journal.pone.0310054 (PMC11379216; doi:10.1371/journal.pone.0310054)
Supplement: S1 File — (DOCX) [file pone.0310054.s004.docx]

**Zn-BTC Cu-BTC Fe-BTC**

**2 Theta Counts 2 Theta Counts 2 Theta Counts**

| 10.34 | 2382.8 |  | 10.34 | 1411.2 |  | 10.34 | 687.4 |
| --- | --- | --- | --- | --- | --- | --- | --- |
| 10.39 | 1734.6 |  | 10.39 | 1472.8 |  | 10.39 | 940.8 |
| 10.44 | 1738.8 |  | 10.44 | 1467.2 |  | 10.44 | 911.4 |
| 10.49 | 1345.4 |  | 10.49 | 1555.4 |  | 10.49 | 1010.8 |
| 10.54 | 940.8 |  | 10.54 | 1498 |  | 10.54 | 1006.6 |
| 10.59 | 928.2 |  | 10.59 | 1512 |  | 10.59 | 992.6 |
| 10.64 | 926.8 |  | 10.64 | 1516.2 |  | 10.64 | 950.6 |
| 10.69 | 786.8 |  | 10.69 | 1583.4 |  | 10.69 | 922.6 |
| 10.74 | 770 |  | 10.74 | 1556.8 |  | 10.74 | 925.4 |
| 10.79 | 992.6 |  | 10.79 | 1486.8 |  | 10.79 | 854 |
| 10.84 | 995.4 |  | 10.84 | 1484 |  | 10.84 | 894.6 |
| 10.89 | 1110.2 |  | 10.89 | 1496.6 |  | 10.89 | 938 |
| 10.94 | 1176 |  | 10.94 | 1654.8 |  | 10.94 | 939.4 |
| 10.99 | 1247.4 |  | 10.99 | 1929.2 |  | 10.99 | 795.2 |
| 11.04 | 1248.8 |  | 11.04 | 1930.6 |  | 11.04 | 814.8 |
| 11.09 | 992.6 |  | 11.09 | 2298.8 |  | 11.09 | 838.6 |
| 11.14 | 870.8 |  | 11.14 | 2787.4 |  | 11.14 | 840 |
| 11.19 | 812 |  | 11.19 | 3137.4 |  | 11.19 | 770 |
| 11.24 | 813.4 |  | 11.24 | 3134.6 |  | 11.24 | 662.2 |
| 11.29 | 656.6 |  | 11.29 | 3852.8 |  | 11.29 | 730.8 |
| 11.34 | 560 |  | 11.34 | 4383.4 |  | 11.34 | 729.4 |
| 11.39 | 630 |  | 11.39 | 5727.4 |  | 11.39 | 771.4 |
| 11.44 | 630 |  | 11.44 | 5728.8 |  | 11.44 | 627.2 |
| 11.49 | 560 |  | 11.49 | 7365.4 |  | 11.49 | 670.6 |
| 11.54 | 585.2 |  | 11.54 | 9620.8 |  | 11.54 | 670.6 |
| 11.59 | 613.2 |  | 11.59 | 12041.4 |  | 11.59 | 557.2 |
| 11.64 | 616 |  | 11.64 | 12042.8 |  | 11.64 | 532 |
| 11.69 | 576.8 |  | 11.69 | 12730.2 |  | 11.69 | 533.4 |
| 11.74 | 614.6 |  | 11.74 | 11491.2 |  | 11.74 | 534.8 |
| 11.79 | 534.8 |  | 11.79 | 7534.8 |  | 11.79 | 508.2 |
| 11.84 | 534.8 |  | 11.84 | 7532 |  | 11.84 | 504 |
| 11.89 | 558.6 |  | 11.89 | 4198.6 |  | 11.89 | 474.6 |
| 11.94 | 505.4 |  | 11.94 | 2508.8 |  | 11.94 | 473.2 |
| 11.99 | 618.8 |  | 11.99 | 2060.8 |  | 11.99 | 548.8 |
| 12.04 | 620.2 |  | 12.04 | 2060.8 |  | 12.04 | 562.8 |
| 12.09 | 618.8 |  | 12.09 | 1653.4 |  | 12.09 | 477.4 |
| 12.14 | 617.4 |  | 12.14 | 1694 |  | 12.14 | 473.2 |
| 12.19 | 618.8 |  | 12.19 | 1569.4 |  | 12.19 | 532 |
| 12.24 | 620.2 |  | 12.24 | 1569.4 |  | 12.24 | 515.2 |
| 12.29 | 548.8 |  | 12.29 | 1734.6 |  | 12.29 | 435.4 |
| 12.34 | 571.2 |  | 12.34 | 1710.8 |  | 12.34 | 431.2 |
| 12.39 | 590.8 |  | 12.39 | 1635.2 |  | 12.39 | 446.6 |
| 12.44 | 589.4 |  | 12.44 | 1638 |  | 12.44 | 464.8 |
| 12.49 | 572.6 |  | 12.49 | 1723.4 |  | 12.49 | 407.4 |
| 12.54 | 618.8 |  | 12.54 | 1736 |  | 12.54 | 410.2 |
| 12.59 | 602 |  | 12.59 | 1638 |  | 12.59 | 404.6 |
| 12.64 | 603.4 |  | 12.64 | 1639.4 |  | 12.64 | 491.4 |
| 12.69 | 574 |  | 12.69 | 1780.8 |  | 12.69 | 438.2 |
| 12.74 | 646.8 |  | 12.74 | 1653.4 |  | 12.74 | 434 |
| 12.79 | 644 |  | 12.79 | 1831.2 |  | 12.79 | 420 |
| 12.84 | 644 |  | 12.84 | 1832.6 |  | 12.84 | 491.4 |
| 12.89 | 614.6 |  | 12.89 | 2018.8 |  | 12.89 | 421.4 |
| 12.94 | 684.6 |  | 12.94 | 1976.8 |  | 12.94 | 424.2 |
| 12.99 | 683.2 |  | 12.99 | 2465.4 |  | 12.99 | 518 |
| 13.04 | 683.2 |  | 13.04 | 2465.4 |  | 13.04 | 404.6 |
| 13.09 | 687.4 |  | 13.09 | 2657.2 |  | 13.09 | 407.4 |
| 13.14 | 659.4 |  | 13.14 | 3024 |  | 13.14 | 406 |
| 13.19 | 644 |  | 13.19 | 3022.6 |  | 13.19 | 508.2 |
| 13.24 | 648.2 |  | 13.24 | 3669.4 |  | 13.24 | 434 |
| 13.29 | 824.6 |  | 13.29 | 5139.4 |  | 13.29 | 407.4 |
| 13.34 | 760.2 |  | 13.34 | 6678 |  | 13.34 | 408.8 |
| 13.39 | 718.2 |  | 13.39 | 6676.6 |  | 13.39 | 407.4 |
| 13.44 | 712.6 |  | 13.44 | 8163.4 |  | 13.44 | 421.4 |
| 13.49 | 798 |  | 13.49 | 8527.4 |  | 13.49 | 446.6 |
| 13.54 | 854 |  | 13.54 | 7350 |  | 13.54 | 449.4 |
| 13.59 | 858.2 |  | 13.59 | 7352.8 |  | 13.59 | 421.4 |
| 13.64 | 851.2 |  | 13.64 | 5304.6 |  | 13.64 | 424.2 |
| 13.69 | 844.2 |  | 13.69 | 3273.2 |  | 13.69 | 529.2 |
| 13.74 | 842.8 |  | 13.74 | 2395.4 |  | 13.74 | 534.8 |
| 13.79 | 956.2 |  | 13.79 | 2394 |  | 13.79 | 434 |
| 13.84 | 953.4 |  | 13.84 | 1978.2 |  | 13.84 | 432.6 |
| 13.89 | 802.2 |  | 13.89 | 1958.6 |  | 13.89 | 529.2 |
| 13.94 | 828.8 |  | 13.94 | 1894.2 |  | 13.94 | 534.8 |
| 13.99 | 827.4 |  | 13.99 | 1894.2 |  | 13.99 | 448 |
| 14.04 | 926.8 |  | 14.04 | 1958.6 |  | 14.04 | 546 |
| 14.09 | 907.2 |  | 14.09 | 2070.6 |  | 14.09 | 606.2 |
| 14.14 | 954.8 |  | 14.14 | 1947.4 |  | 14.14 | 602 |
| 14.19 | 953.4 |  | 14.19 | 1944.6 |  | 14.19 | 519.4 |
| 14.24 | 952 |  | 14.24 | 1943.2 |  | 14.24 | 600.6 |
| 14.29 | 1005.2 |  | 14.29 | 2146.2 |  | 14.29 | 505.4 |
| 14.34 | 1110.2 |  | 14.34 | 2072 |  | 14.34 | 502.6 |
| 14.39 | 1107.4 |  | 14.39 | 2069.2 |  | 14.39 | 548.8 |
| 14.44 | 1346.8 |  | 14.44 | 2114 |  | 14.44 | 492.8 |
| 14.49 | 1401.4 |  | 14.49 | 2408 |  | 14.49 | 502.6 |
| 14.54 | 1596 |  | 14.54 | 2531.2 |  | 14.54 | 506.8 |
| 14.59 | 1596 |  | 14.59 | 2532.6 |  | 14.59 | 576.8 |
| 14.64 | 1554 |  | 14.64 | 2756.6 |  | 14.64 | 548.8 |
| 14.69 | 1495.2 |  | 14.69 | 3026.8 |  | 14.69 | 548.8 |
| 14.74 | 1288 |  | 14.74 | 3067.4 |  | 14.74 | 544.6 |
| 14.79 | 1288 |  | 14.79 | 3068.8 |  | 14.79 | 516.6 |
| 14.84 | 1188.6 |  | 14.84 | 3207.4 |  | 14.84 | 562.8 |
| 14.89 | 1318.8 |  | 14.89 | 3148.6 |  | 14.89 | 502.6 |
| 14.94 | 1498 |  | 14.94 | 3672.2 |  | 14.94 | 506.8 |
| 14.99 | 1498 |  | 14.99 | 3669.4 |  | 14.99 | 544.6 |
| 15.04 | 1932 |  | 15.04 | 4016.6 |  | 15.04 | 504 |
| 15.09 | 2311.4 |  | 15.09 | 4008.2 |  | 15.09 | 508.2 |
| 15.14 | 2350.6 |  | 15.14 | 3532.2 |  | 15.14 | 504 |
| 15.19 | 2350.6 |  | 15.19 | 3532.2 |  | 15.19 | 519.4 |
| 15.24 | 2461.2 |  | 15.24 | 2868.6 |  | 15.24 | 474.6 |
| 15.29 | 1831.2 |  | 15.29 | 2420.6 |  | 15.29 | 506.8 |
| 15.34 | 1583.4 |  | 15.34 | 2268 |  | 15.34 | 505.4 |
| 15.39 | 1583.4 |  | 15.39 | 2268 |  | 15.39 | 462 |
| 15.44 | 1388.8 |  | 15.44 | 2076.2 |  | 15.44 | 477.4 |
| 15.49 | 1444.8 |  | 15.49 | 2098.6 |  | 15.49 | 463.4 |
| 15.54 | 1398.6 |  | 15.54 | 1919.4 |  | 15.54 | 462 |
| 15.59 | 1402.8 |  | 15.59 | 1918 |  | 15.59 | 446.6 |
| 15.64 | 1656.2 |  | 15.64 | 2112.6 |  | 15.64 | 501.2 |
| 15.69 | 1792 |  | 15.69 | 2048.2 |  | 15.69 | 436.8 |
| 15.74 | 1736 |  | 15.74 | 2088.8 |  | 15.74 | 432.6 |
| 15.79 | 1737.4 |  | 15.79 | 2086 |  | 15.79 | 424.2 |
| 15.84 | 1495.2 |  | 15.84 | 2156 |  | 15.84 | 476 |
| 15.89 | 1418.2 |  | 15.89 | 2202.2 |  | 15.89 | 378 |
| 15.94 | 1233.4 |  | 15.94 | 2244.2 |  | 15.94 | 376.6 |
| 15.99 | 1236.2 |  | 15.99 | 2241.4 |  | 15.99 | 506.8 |
| 16.04 | 1134 |  | 16.04 | 2167.2 |  | 16.04 | 490 |
| 16.09 | 1202.6 |  | 16.09 | 2468.2 |  | 16.09 | 432.6 |
| 16.14 | 1230.6 |  | 16.14 | 2618 |  | 16.14 | 431.2 |
| 16.19 | 1229.2 |  | 16.19 | 2616.6 |  | 16.19 | 488.6 |
| 16.24 | 1094.8 |  | 16.24 | 2518.6 |  | 16.24 | 505.4 |
| 16.29 | 1079.4 |  | 16.29 | 3022.6 |  | 16.29 | 571.2 |
| 16.34 | 1131.2 |  | 16.34 | 3515.4 |  | 16.34 | 576.8 |
| 16.39 | 1138.2 |  | 16.39 | 3511.2 |  | 16.39 | 463.4 |
| 16.44 | 1243.2 |  | 16.44 | 3959.2 |  | 16.44 | 562.8 |
| 16.49 | 1292.2 |  | 16.49 | 4272.8 |  | 16.49 | 644 |
| 16.54 | 1288 |  | 16.54 | 4144 |  | 16.54 | 642.6 |
| 16.59 | 1288 |  | 16.59 | 4146.8 |  | 16.59 | 578.2 |
| 16.64 | 1383.2 |  | 16.64 | 3600.8 |  | 16.64 | 700 |
| 16.69 | 1331.4 |  | 16.69 | 2899.4 |  | 16.69 | 697.2 |
| 16.74 | 1334.2 |  | 16.74 | 2580.2 |  | 16.74 | 659.4 |
| 16.79 | 1332.8 |  | 16.79 | 2574.6 |  | 16.79 | 726.6 |
| 16.84 | 1288 |  | 16.84 | 2518.6 |  | 16.84 | 746.2 |
| 16.89 | 1289.4 |  | 16.89 | 2490.6 |  | 16.89 | 739.2 |
| 16.94 | 1400 |  | 16.94 | 2560.6 |  | 16.94 | 855.4 |
| 16.99 | 1401.4 |  | 16.99 | 2562 |  | 16.99 | 855.4 |
| 17.04 | 1428 |  | 17.04 | 2699.2 |  | 17.04 | 698.6 |
| 17.09 | 1516.2 |  | 17.09 | 2661.4 |  | 17.09 | 701.4 |
| 17.14 | 1864.8 |  | 17.14 | 2857.4 |  | 17.14 | 617.4 |
| 17.19 | 1862 |  | 17.19 | 2853.2 |  | 17.19 | 574 |
| 17.24 | 2354.8 |  | 17.24 | 3252.2 |  | 17.24 | 557.2 |
| 17.29 | 2886.8 |  | 17.29 | 3962 |  | 17.29 | 561.4 |
| 17.34 | 3992.8 |  | 17.34 | 4662 |  | 17.34 | 574 |
| 17.39 | 3991.4 |  | 17.39 | 4664.8 |  | 17.39 | 588 |
| 17.44 | 5587.4 |  | 17.44 | 6176.8 |  | 17.44 | 520.8 |
| 17.49 | 8457.4 |  | 17.49 | 6721.4 |  | 17.49 | 522.2 |
| 17.54 | 12069.4 |  | 17.54 | 6060.6 |  | 17.54 | 506.8 |
| 17.59 | 12068 |  | 17.59 | 6066.2 |  | 17.59 | 572.6 |
| 17.64 | 14354.2 |  | 17.64 | 4687.2 |  | 17.64 | 571.2 |
| 17.69 | 13386.8 |  | 17.69 | 3318 |  | 17.69 | 576.8 |
| 17.74 | 10222.8 |  | 17.74 | 2674 |  | 17.74 | 571.2 |
| 17.79 | 10220 |  | 17.79 | 2671.2 |  | 17.79 | 578.2 |
| 17.84 | 8906.8 |  | 17.84 | 2548 |  | 17.84 | 660.8 |
| 17.89 | 7683.2 |  | 17.89 | 2592.8 |  | 17.89 | 660.8 |
| 17.94 | 5702.2 |  | 17.94 | 2531.2 |  | 17.94 | 557.2 |
| 17.99 | 5700.8 |  | 17.99 | 2534 |  | 17.99 | 670.6 |
| 18.04 | 3586.8 |  | 18.04 | 2576 |  | 18.04 | 520.8 |
| 18.09 | 2237.2 |  | 18.09 | 2546.6 |  | 18.09 | 516.6 |
| 18.14 | 2088.8 |  | 18.14 | 2532.6 |  | 18.14 | 659.4 |
| 18.19 | 2084.6 |  | 18.19 | 2534 |  | 18.19 | 642.6 |
| 18.24 | 2172.8 |  | 18.24 | 2492 |  | 18.24 | 604.8 |
| 18.29 | 2517.2 |  | 18.29 | 2522.8 |  | 18.29 | 603.4 |
| 18.34 | 3320.8 |  | 18.34 | 2395.4 |  | 18.34 | 631.4 |
| 18.39 | 3320.8 |  | 18.39 | 2391.2 |  | 18.39 | 604.8 |
| 18.44 | 4856.6 |  | 18.44 | 2520 |  | 18.44 | 627.2 |
| 18.49 | 7446.6 |  | 18.49 | 2632 |  | 18.49 | 634.2 |
| 18.54 | 11048.8 |  | 18.54 | 2914.8 |  | 18.54 | 687.4 |
| 18.59 | 11048.8 |  | 18.59 | 2909.2 |  | 18.59 | 616 |
| 18.64 | 15524.6 |  | 18.64 | 3049.2 |  | 18.64 | 589.4 |
| 18.69 | 18673.2 |  | 18.69 | 3722.6 |  | 18.69 | 589.4 |
| 18.74 | 19541.2 |  | 18.74 | 4438 |  | 18.74 | 614.6 |
| 18.79 | 19544 |  | 18.79 | 4436.6 |  | 18.79 | 669.2 |
| 18.84 | 15556.8 |  | 18.84 | 5334 |  | 18.84 | 578.2 |
| 18.89 | 9982 |  | 18.89 | 6766.2 |  | 18.89 | 572.6 |
| 18.94 | 5251.4 |  | 18.94 | 8680 |  | 18.94 | 687.4 |
| 18.99 | 5251.4 |  | 18.99 | 8680 |  | 18.99 | 561.4 |
| 19.04 | 2731.4 |  | 19.04 | 10459.4 |  | 19.04 | 628.6 |
| 19.09 | 1888.6 |  | 19.09 | 10470.6 |  | 19.09 | 628.6 |
| 19.14 | 1666 |  | 19.14 | 8513.4 |  | 19.14 | 670.6 |
| 19.19 | 1664.6 |  | 19.19 | 8510.6 |  | 19.19 | 656.6 |
| 19.24 | 1621.2 |  | 19.24 | 5755.4 |  | 19.24 | 658 |
| 19.29 | 1584.8 |  | 19.29 | 3682 |  | 19.29 | 662.2 |
| 19.34 | 1542.8 |  | 19.34 | 3134.6 |  | 19.34 | 740.6 |
| 19.39 | 1542.8 |  | 19.39 | 3137.4 |  | 19.39 | 758.8 |
| 19.44 | 1736 |  | 19.44 | 3007.2 |  | 19.44 | 712.6 |
| 19.49 | 1723.4 |  | 19.49 | 2815.4 |  | 19.49 | 712.6 |
| 19.54 | 2003.4 |  | 19.54 | 2704.8 |  | 19.54 | 628.6 |
| 19.59 | 2006.2 |  | 19.59 | 2703.4 |  | 19.59 | 726.6 |
| 19.64 | 2144.8 |  | 19.64 | 2801.4 |  | 19.64 | 728 |
| 19.69 | 2226 |  | 19.69 | 2713.2 |  | 19.69 | 729.4 |
| 19.74 | 2188.2 |  | 19.74 | 2997.4 |  | 19.74 | 700 |
| 19.79 | 2184 |  | 19.79 | 2997.4 |  | 19.79 | 726.6 |
| 19.84 | 2060.8 |  | 19.84 | 3000.2 |  | 19.84 | 746.2 |
| 19.89 | 2342.2 |  | 19.89 | 3206 |  | 19.89 | 740.6 |
| 19.94 | 2770.6 |  | 19.94 | 3473.4 |  | 19.94 | 942.2 |
| 19.99 | 2774.8 |  | 19.99 | 3474.8 |  | 19.99 | 1190 |
| 20.04 | 3109.4 |  | 20.04 | 4032 |  | 20.04 | 1290.8 |
| 20.09 | 3176.6 |  | 20.09 | 4750.2 |  | 20.09 | 1288 |
| 20.14 | 2830.8 |  | 20.14 | 5598.6 |  | 20.14 | 1723.4 |
| 20.19 | 2826.6 |  | 20.19 | 5598.6 |  | 20.19 | 2028.6 |
| 20.24 | 2171.4 |  | 20.24 | 5854.8 |  | 20.24 | 2296 |
| 20.29 | 1916.6 |  | 20.29 | 5378.8 |  | 20.29 | 2296 |
| 20.34 | 1779.4 |  | 20.34 | 4029.2 |  | 20.34 | 2364.6 |
| 20.39 | 1778 |  | 20.39 | 4030.6 |  | 20.39 | 2608.2 |
| 20.44 | 1710.8 |  | 20.44 | 3066 |  | 20.44 | 2462.6 |
| 20.49 | 1695.4 |  | 20.49 | 2774.8 |  | 20.49 | 2466.8 |
| 20.54 | 1583.4 |  | 20.54 | 2688 |  | 20.54 | 2377.2 |
| 20.59 | 1582 |  | 20.59 | 2689.4 |  | 20.59 | 1708 |
| 20.64 | 1402.8 |  | 20.64 | 2717.4 |  | 20.64 | 1078 |
| 20.69 | 1444.8 |  | 20.69 | 2574.6 |  | 20.69 | 1080.8 |
| 20.74 | 1376.2 |  | 20.74 | 2728.6 |  | 20.74 | 784 |
| 20.79 | 1374.8 |  | 20.79 | 2730 |  | 20.79 | 688.8 |
| 20.84 | 1499.4 |  | 20.84 | 2647.4 |  | 20.84 | 674.8 |
| 20.89 | 1486.8 |  | 20.89 | 2759.4 |  | 20.89 | 676.2 |
| 20.94 | 1327.2 |  | 20.94 | 2816.8 |  | 20.94 | 674.8 |
| 20.99 | 1328.6 |  | 20.99 | 2818.2 |  | 20.99 | 550.2 |
| 21.04 | 1677.2 |  | 21.04 | 2703.4 |  | 21.04 | 602 |
| 21.09 | 1706.6 |  | 21.09 | 3012.8 |  | 21.09 | 599.2 |
| 21.14 | 1948.8 |  | 21.14 | 3231.2 |  | 21.14 | 586.6 |
| 21.19 | 1947.4 |  | 21.19 | 3236.8 |  | 21.19 | 600.6 |
| 21.24 | 2186.8 |  | 21.24 | 3554.6 |  | 21.24 | 656.6 |
| 21.29 | 2531.2 |  | 21.29 | 3780 |  | 21.29 | 658 |
| 21.34 | 2520 |  | 21.34 | 3781.4 |  | 21.34 | 715.4 |
| 21.39 | 2517.2 |  | 21.39 | 3812.2 |  | 21.39 | 688.8 |
| 21.44 | 2493.4 |  | 21.44 | 3402 |  | 21.44 | 655.2 |
| 21.49 | 2059.4 |  | 21.49 | 3050.6 |  | 21.49 | 662.2 |
| 21.54 | 1736 |  | 21.54 | 3053.4 |  | 21.54 | 698.6 |
| 21.59 | 1734.6 |  | 21.59 | 3052 |  | 21.59 | 628.6 |
| 21.64 | 1530.2 |  | 21.64 | 2924.6 |  | 21.64 | 701.4 |
| 21.69 | 1579.2 |  | 21.69 | 2727.2 |  | 21.69 | 698.6 |
| 21.74 | 2013.2 |  | 21.74 | 2728.6 |  | 21.74 | 655.2 |
| 21.79 | 2016 |  | 21.79 | 2662.8 |  | 21.79 | 728 |
| 21.84 | 2756.6 |  | 21.84 | 2717.4 |  | 21.84 | 743.4 |
| 21.89 | 3418.8 |  | 21.89 | 2825.2 |  | 21.89 | 739.2 |
| 21.94 | 4608.8 |  | 21.94 | 2828 |  | 21.94 | 702.8 |
| 21.99 | 4608.8 |  | 21.99 | 2882.6 |  | 21.99 | 729.4 |
| 22.04 | 5182.8 |  | 22.04 | 2912 |  | 22.04 | 656.6 |
| 22.09 | 5069.4 |  | 22.09 | 3008.6 |  | 22.09 | 662.2 |
| 22.14 | 3808 |  | 22.14 | 3008.6 |  | 22.14 | 702.8 |
| 22.19 | 3812.2 |  | 22.19 | 2899.4 |  | 22.19 | 714 |
| 22.24 | 2688 |  | 22.24 | 2825.2 |  | 22.24 | 785.4 |
| 22.29 | 1878.8 |  | 22.29 | 3007.2 |  | 22.29 | 786.8 |
| 22.34 | 1877.4 |  | 22.34 | 3011.4 |  | 22.34 | 952 |
| 22.39 | 1554 |  | 22.39 | 2927.4 |  | 22.39 | 796.6 |
| 22.44 | 1444.8 |  | 22.44 | 3022.6 |  | 22.44 | 799.4 |
| 22.49 | 1457.4 |  | 22.49 | 3039.4 |  | 22.49 | 800.8 |
| 22.54 | 1457.4 |  | 22.54 | 3039.4 |  | 22.54 | 953.4 |
| 22.59 | 1274 |  | 22.59 | 2804.2 |  | 22.59 | 968.8 |
| 22.64 | 1430.8 |  | 22.64 | 2868.6 |  | 22.64 | 1054.2 |
| 22.69 | 1412.6 |  | 22.69 | 2843.4 |  | 22.69 | 1050 |
| 22.74 | 1415.4 |  | 22.74 | 2839.2 |  | 22.74 | 1272.6 |
| 22.79 | 1440.6 |  | 22.79 | 2956.8 |  | 22.79 | 1481.2 |
| 22.84 | 1342.6 |  | 22.84 | 2979.2 |  | 22.84 | 1275.4 |
| 22.89 | 1551.2 |  | 22.89 | 3126.2 |  | 22.89 | 1272.6 |
| 22.94 | 1551.2 |  | 22.94 | 3124.8 |  | 22.94 | 1302 |
| 22.99 | 1482.6 |  | 22.99 | 3138.8 |  | 22.99 | 967.4 |
| 23.04 | 1454.6 |  | 23.04 | 3094 |  | 23.04 | 953.4 |
| 23.09 | 1428 |  | 23.09 | 3278.8 |  | 23.09 | 953.4 |
| 23.14 | 1426.6 |  | 23.14 | 3277.4 |  | 23.14 | 856.8 |
| 23.19 | 1342.6 |  | 23.19 | 3420.2 |  | 23.19 | 866.6 |
| 23.24 | 1244.6 |  | 23.24 | 4184.6 |  | 23.24 | 991.2 |
| 23.29 | 1215.2 |  | 23.29 | 4396 |  | 23.29 | 998.2 |
| 23.34 | 1219.4 |  | 23.34 | 4397.4 |  | 23.34 | 925.4 |
| 23.39 | 1258.6 |  | 23.39 | 4452 |  | 23.39 | 914.2 |
| 23.44 | 1430.8 |  | 23.44 | 4298 |  | 23.44 | 841.4 |
| 23.49 | 1439.2 |  | 23.49 | 3710 |  | 23.49 | 840 |
| 23.54 | 1439.2 |  | 23.54 | 3714.2 |  | 23.54 | 894.6 |
| 23.59 | 1467.2 |  | 23.59 | 3320.8 |  | 23.59 | 907.2 |
| 23.64 | 1471.4 |  | 23.64 | 3122 |  | 23.64 | 883.4 |
| 23.69 | 1387.4 |  | 23.69 | 3175.2 |  | 23.69 | 883.4 |
| 23.74 | 1384.6 |  | 23.74 | 3175.2 |  | 23.74 | 908.6 |
| 23.79 | 1499.4 |  | 23.79 | 3008.6 |  | 23.79 | 898.8 |
| 23.84 | 1317.4 |  | 23.84 | 3516.8 |  | 23.84 | 995.4 |
| 23.89 | 1344 |  | 23.89 | 3641.4 |  | 23.89 | 996.8 |
| 23.94 | 1345.4 |  | 23.94 | 3642.8 |  | 23.94 | 844.2 |
| 23.99 | 1290.8 |  | 23.99 | 4116 |  | 23.99 | 883.4 |
| 24.04 | 1414 |  | 24.04 | 4813.2 |  | 24.04 | 966 |
| 24.09 | 1289.4 |  | 24.09 | 5136.6 |  | 24.09 | 964.6 |
| 24.14 | 1285.2 |  | 24.14 | 5135.2 |  | 24.14 | 813.4 |
| 24.19 | 1275.4 |  | 24.19 | 5086.2 |  | 24.19 | 898.8 |
| 24.24 | 1260 |  | 24.24 | 4622.8 |  | 24.24 | 870.8 |
| 24.29 | 1318.8 |  | 24.29 | 4463.2 |  | 24.29 | 872.2 |
| 24.34 | 1314.6 |  | 24.34 | 4464.6 |  | 24.34 | 858.2 |
| 24.39 | 1398.6 |  | 24.39 | 4256 |  | 24.39 | 816.2 |
| 24.44 | 1430.8 |  | 24.44 | 3852.8 |  | 24.44 | 814.8 |
| 24.49 | 1482.6 |  | 24.49 | 3476.2 |  | 24.49 | 809.2 |
| 24.54 | 1486.8 |  | 24.54 | 3472 |  | 24.54 | 743.4 |
| 24.59 | 1488.2 |  | 24.59 | 3262 |  | 24.59 | 828.8 |
| 24.64 | 1681.4 |  | 24.64 | 3120.6 |  | 24.64 | 697.2 |
| 24.69 | 1754.2 |  | 24.69 | 3120.6 |  | 24.69 | 700 |
| 24.74 | 1750 |  | 24.74 | 3123.4 |  | 24.74 | 758.8 |
| 24.79 | 1640.8 |  | 24.79 | 3028.2 |  | 24.79 | 767.2 |
| 24.84 | 1556.8 |  | 24.84 | 2914.8 |  | 24.84 | 782.6 |
| 24.89 | 1362.2 |  | 24.89 | 3178 |  | 24.89 | 781.2 |
| 24.94 | 1362.2 |  | 24.94 | 3176.6 |  | 24.94 | 782.6 |
| 24.99 | 1248.8 |  | 24.99 | 3182.2 |  | 24.99 | 684.6 |
| 25.04 | 1327.2 |  | 25.04 | 3248 |  | 25.04 | 782.6 |
| 25.09 | 1257.2 |  | 25.09 | 3375.4 |  | 25.09 | 785.4 |
| 25.14 | 1258.6 |  | 25.14 | 3372.6 |  | 25.14 | 729.4 |
| 25.19 | 1398.6 |  | 25.19 | 3708.6 |  | 25.19 | 688.8 |
| 25.24 | 1580.6 |  | 25.24 | 4061.4 |  | 25.24 | 684.6 |
| 25.29 | 1681.4 |  | 25.29 | 3854.2 |  | 25.29 | 659.4 |
| 25.34 | 1680 |  | 25.34 | 3848.6 |  | 25.34 | 770 |
| 25.39 | 1892.8 |  | 25.39 | 3644.2 |  | 25.39 | 716.8 |
| 25.44 | 1986.6 |  | 25.44 | 3540.6 |  | 25.44 | 716.8 |
| 25.49 | 1792 |  | 25.49 | 3402 |  | 25.49 | 742 |
| 25.54 | 1792 |  | 25.54 | 3402 |  | 25.54 | 714 |
| 25.59 | 1680 |  | 25.59 | 3417.4 |  | 25.59 | 670.6 |
| 25.64 | 1737.4 |  | 25.64 | 4201.4 |  | 25.64 | 670.6 |
| 25.69 | 1873.2 |  | 25.69 | 4954.6 |  | 25.69 | 798 |
| 25.74 | 1876 |  | 25.74 | 4958.8 |  | 25.74 | 782.6 |
| 25.79 | 2224.6 |  | 25.79 | 6273.4 |  | 25.79 | 785.4 |
| 25.84 | 2634.8 |  | 25.84 | 8485.4 |  | 25.84 | 786.8 |
| 25.89 | 2773.4 |  | 25.89 | 9968 |  | 25.89 | 954.8 |
| 25.94 | 2772 |  | 25.94 | 9972.2 |  | 25.94 | 897.4 |
| 25.99 | 3458 |  | 25.99 | 10119.2 |  | 25.99 | 1079.4 |
| 26.04 | 3838.8 |  | 26.04 | 8202.6 |  | 26.04 | 1076.6 |
| 26.09 | 3711.4 |  | 26.09 | 5630.8 |  | 26.09 | 968.8 |
| 26.14 | 3708.6 |  | 26.14 | 5630.8 |  | 26.14 | 1048.6 |
| 26.19 | 3078.6 |  | 26.19 | 3918.6 |  | 26.19 | 980 |
| 26.24 | 2578.8 |  | 26.24 | 3358.6 |  | 26.24 | 981.4 |
| 26.29 | 2350.6 |  | 26.29 | 3234 |  | 26.29 | 926.8 |
| 26.34 | 2353.4 |  | 26.34 | 3238.2 |  | 26.34 | 842.8 |
| 26.39 | 2573.2 |  | 26.39 | 3238.2 |  | 26.39 | 798 |
| 26.44 | 3274.6 |  | 26.44 | 3140.2 |  | 26.44 | 799.4 |
| 26.49 | 3767.4 |  | 26.49 | 2996 |  | 26.49 | 740.6 |
| 26.54 | 3764.6 |  | 26.54 | 2994.6 |  | 26.54 | 770 |
| 26.59 | 3977.4 |  | 26.59 | 3050.6 |  | 26.59 | 838.6 |
| 26.64 | 3252.2 |  | 26.64 | 3049.2 |  | 26.64 | 838.6 |
| 26.69 | 2536.8 |  | 26.69 | 3152.8 |  | 26.69 | 802.2 |
| 26.74 | 2534 |  | 26.74 | 3150 |  | 26.74 | 814.8 |
| 26.79 | 2311.4 |  | 26.79 | 3082.8 |  | 26.79 | 799.4 |
| 26.84 | 2969.4 |  | 26.84 | 3220 |  | 26.84 | 800.8 |
| 26.89 | 3514 |  | 26.89 | 3224.2 |  | 26.89 | 795.2 |
| 26.94 | 3512.6 |  | 26.94 | 3221.4 |  | 26.94 | 800.8 |
| 26.99 | 4845.4 |  | 26.99 | 3235.4 |  | 26.99 | 884.8 |
| 27.04 | 6848.8 |  | 27.04 | 3490.2 |  | 27.04 | 882 |
| 27.09 | 8066.8 |  | 27.09 | 3474.8 |  | 27.09 | 756 |
| 27.14 | 8062.6 |  | 27.14 | 3472 |  | 27.14 | 852.6 |
| 27.19 | 7936.6 |  | 27.19 | 3278.8 |  | 27.19 | 758.8 |
| 27.24 | 5908 |  | 27.24 | 3112.2 |  | 27.24 | 756 |
| 27.29 | 3474.8 |  | 27.29 | 3248 |  | 27.29 | 880.6 |
| 27.34 | 3470.6 |  | 27.34 | 3245.2 |  | 27.34 | 814.8 |
| 27.39 | 2144.8 |  | 27.39 | 3249.4 |  | 27.39 | 740.6 |
| 27.44 | 1666 |  | 27.44 | 3404.8 |  | 27.44 | 743.4 |
| 27.49 | 1752.8 |  | 27.49 | 3361.4 |  | 27.49 | 826 |
| 27.54 | 1748.6 |  | 27.54 | 3358.6 |  | 27.54 | 900.2 |
| 27.59 | 1804.6 |  | 27.59 | 3693.2 |  | 27.59 | 856.8 |
| 27.64 | 1904 |  | 27.64 | 3768.8 |  | 27.64 | 855.4 |
| 27.69 | 1682.8 |  | 27.69 | 3921.4 |  | 27.69 | 869.4 |
| 27.74 | 1677.2 |  | 27.74 | 3920 |  | 27.74 | 880.6 |
| 27.79 | 1684.2 |  | 27.79 | 3724 |  | 27.79 | 827.4 |
| 27.84 | 1638 |  | 27.84 | 3441.2 |  | 27.84 | 826 |
| 27.89 | 1412.6 |  | 27.89 | 3458 |  | 27.89 | 760.2 |
| 27.94 | 1415.4 |  | 27.94 | 3456.6 |  | 27.94 | 828.8 |
| 27.99 | 1386 |  | 27.99 | 3266.2 |  | 27.99 | 729.4 |
| 28.04 | 1313.2 |  | 28.04 | 3273.2 |  | 28.04 | 725.2 |
| 28.09 | 1276.8 |  | 28.09 | 3109.4 |  | 28.09 | 785.4 |
| 28.14 | 1276.8 |  | 28.14 | 3108 |  | 28.14 | 896 |
| 28.19 | 1387.4 |  | 28.19 | 3235.4 |  | 28.19 | 992.6 |
| 28.24 | 1509.2 |  | 28.24 | 3190.6 |  | 28.24 | 994 |
| 28.29 | 1862 |  | 28.29 | 3178 |  | 28.29 | 1148 |
| 28.34 | 1862 |  | 28.34 | 3180.8 |  | 28.34 | 1442 |
| 28.39 | 2452.8 |  | 28.39 | 3304 |  | 28.39 | 1484 |
| 28.44 | 3571.4 |  | 28.44 | 3484.6 |  | 28.44 | 1482.6 |
| 28.49 | 4692.8 |  | 28.49 | 3866.8 |  | 28.49 | 1390.2 |
| 28.54 | 4691.4 |  | 28.54 | 3868.2 |  | 28.54 | 1402.8 |
| 28.59 | 5250 |  | 28.59 | 4386.2 |  | 28.59 | 1106 |
| 28.64 | 5126.8 |  | 28.64 | 4719.4 |  | 28.64 | 1108.8 |
| 28.69 | 4328.8 |  | 28.69 | 5040 |  | 28.69 | 1006.6 |
| 28.74 | 4328.8 |  | 28.74 | 5040 |  | 28.74 | 894.6 |
| 28.79 | 3178 |  | 28.79 | 4674.6 |  | 28.79 | 837.2 |
| 28.84 | 2170 |  | 28.84 | 4202.8 |  | 28.84 | 844.2 |
| 28.89 | 1706.6 |  | 28.89 | 3656.8 |  | 28.89 | 852.6 |
| 28.94 | 1705.2 |  | 28.94 | 3654 |  | 28.94 | 926.8 |
| 28.99 | 1439.2 |  | 28.99 | 3753.4 |  | 28.99 | 996.8 |
| 29.04 | 1373.4 |  | 29.04 | 4089.4 |  | 29.04 | 996.8 |
| 29.09 | 1454.6 |  | 29.09 | 4901.4 |  | 29.09 | 1258.6 |
| 29.14 | 1453.2 |  | 29.14 | 4904.2 |  | 29.14 | 1608.6 |
| 29.19 | 1764 |  | 29.19 | 6302.8 |  | 29.19 | 1850.8 |
| 29.24 | 2296 |  | 29.24 | 7543.2 |  | 29.24 | 1845.2 |
| 29.29 | 2829.4 |  | 29.29 | 7936.6 |  | 29.29 | 1597.4 |
| 29.34 | 2832.2 |  | 29.34 | 7942.2 |  | 29.34 | 1204 |
| 29.39 | 2912 |  | 29.39 | 7418.6 |  | 29.39 | 967.4 |
| 29.44 | 2786 |  | 29.44 | 5828.2 |  | 29.44 | 968.8 |
| 29.49 | 2480.8 |  | 29.49 | 4296.6 |  | 29.49 | 870.8 |
| 29.54 | 2476.6 |  | 29.54 | 4298 |  | 29.54 | 886.2 |
| 29.59 | 2030 |  | 29.59 | 3472 |  | 29.59 | 922.6 |
| 29.64 | 1906.8 |  | 29.64 | 3106.6 |  | 29.64 | 922.6 |
| 29.69 | 2184 |  | 29.69 | 3110.8 |  | 29.69 | 1066.8 |
| 29.74 | 2185.4 |  | 29.74 | 3332 |  | 29.74 | 1285.2 |
| 29.79 | 2256.8 |  | 29.79 | 3218.6 |  | 29.79 | 1160.6 |
| 29.84 | 2662.8 |  | 29.84 | 3123.4 |  | 29.84 | 1160.6 |
| 29.89 | 2601.2 |  | 29.89 | 3123.4 |  | 29.89 | 1064 |
| 29.94 | 2602.6 |  | 29.94 | 3346 |  | 29.94 | 758.8 |
| 29.99 | 2212 |  | 29.99 | 3292.8 |  | 29.99 | 578.2 |
| 30.04 | 1765.4 |  | 30.04 | 3343.2 |  | 30.04 | 575.4 |
| 30.09 | 1412.6 |  | 30.09 | 3343.2 |  | 30.09 | 603.4 |
| 30.14 | 1414 |  | 30.14 | 3511.2 |  | 30.14 | 562.8 |
| 30.19 | 1314.6 |  | 30.19 | 3655.4 |  | 30.19 | 502.6 |
| 30.24 | 1190 |  | 30.24 | 4190.2 |  | 30.24 | 501.2 |
| 30.29 | 1346.8 |  | 30.29 | 4184.6 |  | 30.29 | 501.2 |
| 30.34 | 1348.2 |  | 30.34 | 4242 |  | 30.34 | 491.4 |
| 30.39 | 1356.6 |  | 30.39 | 4172 |  | 30.39 | 592.2 |
| 30.44 | 1426.6 |  | 30.44 | 3684.8 |  | 30.44 | 589.4 |
| 30.49 | 1458.8 |  | 30.49 | 3680.6 |  | 30.49 | 508.2 |
| 30.54 | 1457.4 |  | 30.54 | 3319.4 |  | 30.54 | 519.4 |
| 30.59 | 1272.6 |  | 30.59 | 3119.2 |  | 30.59 | 519.4 |
| 30.64 | 1402.8 |  | 30.64 | 3262 |  | 30.64 | 516.6 |
| 30.69 | 1348.2 |  | 30.69 | 3264.8 |  | 30.69 | 530.6 |
| 30.74 | 1344 |  | 30.74 | 3616.2 |  | 30.74 | 448 |
| 30.79 | 1360.8 |  | 30.79 | 3822 |  | 30.79 | 589.4 |
| 30.84 | 1355.2 |  | 30.84 | 3792.6 |  | 30.84 | 585.2 |
| 30.89 | 1360.8 |  | 30.89 | 3796.8 |  | 30.89 | 546 |
| 30.94 | 1304.8 |  | 30.94 | 3782.8 |  | 30.94 | 490 |
| 30.99 | 1348.2 |  | 30.99 | 3679.2 |  | 30.99 | 502.6 |
| 31.04 | 1262.8 |  | 31.04 | 3542 |  | 31.04 | 504 |
| 31.09 | 1258.6 |  | 31.09 | 3540.6 |  | 31.09 | 478.8 |
| 31.14 | 1275.4 |  | 31.14 | 3306.8 |  | 31.14 | 462 |
| 31.19 | 1345.4 |  | 31.19 | 3193.4 |  | 31.19 | 576.8 |
| 31.24 | 1482.6 |  | 31.24 | 3138.8 |  | 31.24 | 572.6 |
| 31.29 | 1484 |  | 31.29 | 3133.2 |  | 31.29 | 448 |
| 31.34 | 1656.2 |  | 31.34 | 3194.8 |  | 31.34 | 574 |
| 31.39 | 1664.6 |  | 31.39 | 3179.4 |  | 31.39 | 546 |
| 31.44 | 1835.4 |  | 31.44 | 3049.2 |  | 31.44 | 543.2 |
| 31.49 | 1836.8 |  | 31.49 | 3056.2 |  | 31.49 | 603.4 |
| 31.54 | 1708 |  | 31.54 | 3179.4 |  | 31.54 | 550.2 |
| 31.59 | 1734.6 |  | 31.59 | 3554.6 |  | 31.59 | 516.6 |
| 31.64 | 1890 |  | 31.64 | 3875.2 |  | 31.64 | 520.8 |
| 31.69 | 1890 |  | 31.69 | 3880.8 |  | 31.69 | 534.8 |
| 31.74 | 1794.8 |  | 31.74 | 4202.8 |  | 31.74 | 518 |
| 31.79 | 1762.6 |  | 31.79 | 4690 |  | 31.79 | 558.6 |
| 31.84 | 1570.8 |  | 31.84 | 4299.4 |  | 31.84 | 561.4 |
| 31.89 | 1568 |  | 31.89 | 4296.6 |  | 31.89 | 631.4 |
| 31.94 | 1289.4 |  | 31.94 | 4046 |  | 31.94 | 617.4 |
| 31.99 | 1346.8 |  | 31.99 | 3820.6 |  | 31.99 | 533.4 |
| 32.04 | 1208.2 |  | 32.04 | 3276 |  | 32.04 | 530.6 |
| 32.09 | 1202.6 |  | 32.09 | 3274.6 |  | 32.09 | 645.4 |
| 32.14 | 1342.6 |  | 32.14 | 2927.4 |  | 32.14 | 590.8 |
| 32.19 | 1390.2 |  | 32.19 | 3036.6 |  | 32.19 | 546 |
| 32.24 | 1565.2 |  | 32.24 | 3320.8 |  | 32.24 | 547.4 |
| 32.29 | 1566.6 |  | 32.29 | 3316.6 |  | 32.29 | 562.8 |
| 32.34 | 1908.2 |  | 32.34 | 3203.2 |  | 32.34 | 522.2 |
| 32.39 | 2003.4 |  | 32.39 | 3024 |  | 32.39 | 534.8 |
| 32.44 | 2769.2 |  | 32.44 | 3168.2 |  | 32.44 | 536.2 |
| 32.49 | 2769.2 |  | 32.49 | 3168.2 |  | 32.49 | 544.6 |
| 32.54 | 3221.4 |  | 32.54 | 3192 |  | 32.54 | 478.8 |
| 32.59 | 3819.2 |  | 32.59 | 3091.2 |  | 32.59 | 571.2 |
| 32.64 | 3978.8 |  | 32.64 | 2857.4 |  | 32.64 | 572.6 |
| 32.69 | 3980.2 |  | 32.69 | 2854.6 |  | 32.69 | 550.2 |
| 32.74 | 3406.2 |  | 32.74 | 2958.2 |  | 32.74 | 586.6 |
| 32.79 | 3064.6 |  | 32.79 | 3098.2 |  | 32.79 | 631.4 |
| 32.84 | 2154.6 |  | 32.84 | 2938.6 |  | 32.84 | 631.4 |
| 32.89 | 2154.6 |  | 32.89 | 2938.6 |  | 32.89 | 558.6 |
| 32.94 | 1890 |  | 32.94 | 3026.8 |  | 32.94 | 672 |
| 32.99 | 1874.6 |  | 32.99 | 3248 |  | 32.99 | 674.8 |
| 33.04 | 2504.6 |  | 33.04 | 3022.6 |  | 33.04 | 672 |
| 33.09 | 2506 |  | 33.09 | 3026.8 |  | 33.09 | 683.2 |
| 33.14 | 3123.4 |  | 33.14 | 3106.6 |  | 33.14 | 585.2 |
| 33.19 | 4271.4 |  | 33.19 | 3389.4 |  | 33.19 | 562.8 |
| 33.24 | 5247.2 |  | 33.24 | 3420.2 |  | 33.24 | 562.8 |
| 33.29 | 5250 |  | 33.29 | 3420.2 |  | 33.29 | 560 |
| 33.34 | 5114.2 |  | 33.34 | 3511.2 |  | 33.34 | 502.6 |
| 33.39 | 4618.6 |  | 33.39 | 3332 |  | 33.39 | 644 |
| 33.44 | 3235.4 |  | 33.44 | 3138.8 |  | 33.44 | 644 |
| 33.49 | 3236.8 |  | 33.49 | 3137.4 |  | 33.49 | 670.6 |
| 33.54 | 2254 |  | 33.54 | 3235.4 |  | 33.54 | 823.2 |
| 33.59 | 1778 |  | 33.59 | 3334.8 |  | 33.59 | 869.4 |
| 33.64 | 1960 |  | 33.64 | 3389.4 |  | 33.64 | 866.6 |
| 33.69 | 1961.4 |  | 33.69 | 3389.4 |  | 33.69 | 771.4 |
| 33.74 | 2660 |  | 33.74 | 3654 |  | 33.74 | 715.4 |
| 33.79 | 3274.6 |  | 33.79 | 4118.8 |  | 33.79 | 715.4 |
| 33.84 | 3781.4 |  | 33.84 | 4032 |  | 33.84 | 628.6 |
| 33.89 | 3780 |  | 33.89 | 4032 |  | 33.89 | 576.8 |
| 33.94 | 3334.8 |  | 33.94 | 3949.4 |  | 33.94 | 487.2 |
| 33.99 | 2658.6 |  | 33.99 | 4032 |  | 33.99 | 492.8 |
| 34.04 | 1906.8 |  | 34.04 | 3624.6 |  | 34.04 | 516.6 |
| 34.09 | 1904 |  | 34.09 | 3624.6 |  | 34.09 | 578.2 |
| 34.14 | 1500.8 |  | 34.14 | 3420.2 |  | 34.14 | 534.8 |
| 34.19 | 1299.2 |  | 34.19 | 3220 |  | 34.19 | 534.8 |
| 34.24 | 1230.6 |  | 34.24 | 3106.6 |  | 34.24 | 543.2 |
| 34.29 | 1233.4 |  | 34.29 | 3110.8 |  | 34.29 | 544.6 |
| 34.34 | 1218 |  | 34.34 | 3306.8 |  | 34.34 | 562.8 |
| 34.39 | 1191.4 |  | 34.39 | 3357.2 |  | 34.39 | 562.8 |
| 34.44 | 1342.6 |  | 34.44 | 3529.4 |  | 34.44 | 532 |
| 34.49 | 1344 |  | 34.49 | 3530.8 |  | 34.49 | 505.4 |
| 34.54 | 1401.4 |  | 34.54 | 3640 |  | 34.54 | 478.8 |
| 34.59 | 1286.6 |  | 34.59 | 4114.6 |  | 34.59 | 474.6 |
| 34.64 | 1524.6 |  | 34.64 | 4088 |  | 34.64 | 418.6 |
| 34.69 | 1526 |  | 34.69 | 4089.4 |  | 34.69 | 348.6 |
| 34.74 | 1516.2 |  | 34.74 | 3980.2 |  | 34.74 | 450.8 |
| 34.79 | 1766.8 |  | 34.79 | 4006.8 |  | 34.79 | 450.8 |
| 34.84 | 1654.8 |  | 34.84 | 3795.4 |  | 34.84 | 394.8 |
| 34.89 | 1650.6 |  | 34.89 | 3796.8 |  | 34.89 | 407.4 |
| 34.94 | 1846.6 |  | 34.94 | 4142.6 |  | 34.94 | 434 |
| 34.99 | 1972.6 |  | 34.99 | 4648 |  | 34.99 | 434 |
| 35.04 | 2818.2 |  | 35.04 | 5332.6 |  | 35.04 | 424.2 |
| 35.09 | 2814 |  | 35.09 | 5331.2 |  | 35.09 | 434 |
| 35.14 | 3416 |  | 35.14 | 6539.4 |  | 35.14 | 418.6 |
| 35.19 | 3711.4 |  | 35.19 | 6745.2 |  | 35.19 | 422.8 |
| 35.24 | 3525.2 |  | 35.24 | 6332.2 |  | 35.24 | 431.2 |
| 35.29 | 3529.4 |  | 35.29 | 6329.4 |  | 35.29 | 432.6 |
| 35.34 | 3236.8 |  | 35.34 | 5601.4 |  | 35.34 | 460.6 |
| 35.39 | 3196.2 |  | 35.39 | 4480 |  | 35.39 | 463.4 |
| 35.44 | 3882.2 |  | 35.44 | 3988.6 |  | 35.44 | 618.8 |
| 35.49 | 3879.4 |  | 35.49 | 3991.4 |  | 35.49 | 712.6 |
| 35.54 | 5164.6 |  | 35.54 | 3374 |  | 35.54 | 730.8 |
| 35.59 | 5836.6 |  | 35.59 | 3066 |  | 35.59 | 730.8 |
| 35.64 | 5542.6 |  | 35.64 | 3054.8 |  | 35.64 | 656.6 |
| 35.69 | 5546.8 |  | 35.69 | 3050.6 |  | 35.69 | 586.6 |
| 35.74 | 4174.8 |  | 35.74 | 3218.6 |  | 35.74 | 462 |
| 35.79 | 2630.6 |  | 35.79 | 3249.4 |  | 35.79 | 464.8 |
| 35.84 | 1874.6 |  | 35.84 | 2914.8 |  | 35.84 | 474.6 |
| 35.89 | 1874.6 |  | 35.89 | 2914.8 |  | 35.89 | 438.2 |
| 35.94 | 1635.2 |  | 35.94 | 3165.4 |  | 35.94 | 445.2 |
| 35.99 | 1891.4 |  | 35.99 | 3040.8 |  | 35.99 | 449.4 |
| 36.04 | 2153.2 |  | 36.04 | 3024 |  | 36.04 | 421.4 |
| 36.09 | 2153.2 |  | 36.09 | 3021.2 |  | 36.09 | 452.2 |
| 36.14 | 2396.8 |  | 36.14 | 2998.8 |  | 36.14 | 502.6 |
| 36.19 | 2602.6 |  | 36.19 | 2979.2 |  | 36.19 | 502.6 |
| 36.24 | 2364.6 |  | 36.24 | 3039.4 |  | 36.24 | 575.4 |
| 36.29 | 2364.6 |  | 36.29 | 3038 |  | 36.29 | 550.2 |
| 36.34 | 2006.2 |  | 36.34 | 3304 |  | 36.34 | 492.8 |
| 36.39 | 1552.6 |  | 36.39 | 3152.8 |  | 36.39 | 488.6 |
| 36.44 | 1388.8 |  | 36.44 | 3400.6 |  | 36.44 | 438.2 |
| 36.49 | 1386 |  | 36.49 | 3403.4 |  | 36.49 | 460.6 |
| 36.54 | 1360.8 |  | 36.54 | 3390.8 |  | 36.54 | 474.6 |
| 36.59 | 1442 |  | 36.59 | 3402 |  | 36.59 | 474.6 |
| 36.64 | 1566.6 |  | 36.64 | 3557.4 |  | 36.64 | 490 |
| 36.69 | 1568 |  | 36.69 | 3558.8 |  | 36.69 | 446.6 |
| 36.74 | 1440.6 |  | 36.74 | 3542 |  | 36.74 | 474.6 |
| 36.79 | 1317.4 |  | 36.79 | 3389.4 |  | 36.79 | 477.4 |
| 36.84 | 1230.6 |  | 36.84 | 3210.2 |  | 36.84 | 393.4 |
| 36.89 | 1229.2 |  | 36.89 | 3210.2 |  | 36.89 | 459.2 |
| 36.94 | 1162 |  | 36.94 | 3021.2 |  | 36.94 | 435.4 |
| 36.99 | 1192.8 |  | 36.99 | 3021.2 |  | 36.99 | 434 |
| 37.04 | 1299.2 |  | 37.04 | 3106.6 |  | 37.04 | 492.8 |
| 37.09 | 1304.8 |  | 37.09 | 3108 |  | 37.09 | 435.4 |
| 37.14 | 1346.8 |  | 37.14 | 2941.4 |  | 37.14 | 520.8 |
| 37.19 | 1694 |  | 37.19 | 3151.4 |  | 37.19 | 520.8 |
| 37.24 | 1986.6 |  | 37.24 | 3106.6 |  | 37.24 | 478.8 |
| 37.29 | 1985.2 |  | 37.29 | 3106.6 |  | 37.29 | 460.6 |
| 37.34 | 2342.2 |  | 37.34 | 3162.6 |  | 37.34 | 502.6 |
| 37.39 | 2202.2 |  | 37.39 | 3224.2 |  | 37.39 | 502.6 |
| 37.44 | 2086 |  | 37.44 | 3248 |  | 37.44 | 463.4 |
| 37.49 | 2088.8 |  | 37.49 | 3249.4 |  | 37.49 | 420 |
| 37.54 | 1821.4 |  | 37.54 | 3151.4 |  | 37.54 | 448 |
| 37.59 | 1387.4 |  | 37.59 | 3375.4 |  | 37.59 | 450.8 |
| 37.64 | 1106 |  | 37.64 | 3319.4 |  | 37.64 | 459.2 |
| 37.69 | 1108.8 |  | 37.69 | 3319.4 |  | 37.69 | 436.8 |
| 37.74 | 1094.8 |  | 37.74 | 3655.4 |  | 37.74 | 417.2 |
| 37.79 | 1094.8 |  | 37.79 | 3924.2 |  | 37.79 | 421.4 |
| 37.84 | 1134 |  | 37.84 | 3950.8 |  | 37.84 | 420 |
| 37.89 | 1138.2 |  | 37.89 | 3945.2 |  | 37.89 | 450.8 |
| 37.94 | 1257.2 |  | 37.94 | 3641.4 |  | 37.94 | 452.2 |
| 37.99 | 1432.2 |  | 37.99 | 3404.8 |  | 37.99 | 448 |
| 38.04 | 1510.6 |  | 38.04 | 3402 |  | 38.04 | 449.4 |
| 38.09 | 1512 |  | 38.09 | 3304 |  | 38.09 | 421.4 |
| 38.14 | 1500.8 |  | 38.14 | 3084.2 |  | 38.14 | 396.2 |
| 38.19 | 1400 |  | 38.19 | 3066 |  | 38.19 | 393.4 |
| 38.24 | 1401.4 |  | 38.24 | 3063.2 |  | 38.24 | 378 |
| 38.29 | 1401.4 |  | 38.29 | 3080 |  | 38.29 | 392 |
| 38.34 | 1484 |  | 38.34 | 3273.2 |  | 38.34 | 407.4 |
| 38.39 | 1387.4 |  | 38.39 | 3236.8 |  | 38.39 | 404.6 |
| 38.44 | 1443.4 |  | 38.44 | 3236.8 |  | 38.44 | 408.8 |
| 38.49 | 1443.4 |  | 38.49 | 3138.8 |  | 38.49 | 574 |
| 38.54 | 1412.6 |  | 38.54 | 3319.4 |  | 38.54 | 574 |
| 38.59 | 1500.8 |  | 38.59 | 3361.4 |  | 38.59 | 575.4 |
| 38.64 | 1468.6 |  | 38.64 | 3364.2 |  | 38.64 | 716.8 |
| 38.69 | 1472.8 |  | 38.69 | 3318 |  | 38.69 | 788.2 |
| 38.74 | 1526 |  | 38.74 | 3540.6 |  | 38.74 | 938 |
| 38.79 | 1369.2 |  | 38.79 | 3962 |  | 38.79 | 938 |
| 38.84 | 1485.4 |  | 38.84 | 3960.6 |  | 38.84 | 896 |
| 38.89 | 1486.8 |  | 38.89 | 4673.2 |  | 38.89 | 690.2 |
| 38.94 | 1934.8 |  | 38.94 | 4970 |  | 38.94 | 534.8 |
| 38.99 | 2034.2 |  | 38.99 | 6256.6 |  | 38.99 | 533.4 |
| 39.04 | 2032.8 |  | 39.04 | 6260.8 |  | 39.04 | 502.6 |
| 39.09 | 2450 |  | 39.09 | 6913.2 |  | 39.09 | 516.6 |
| 39.14 | 2368.8 |  | 39.14 | 6892.2 |  | 39.14 | 487.2 |
| 39.19 | 1944.6 |  | 39.19 | 6427.4 |  | 39.19 | 491.4 |
| 39.24 | 1950.2 |  | 39.24 | 6427.4 |  | 39.24 | 364 |
| 39.29 | 1555.4 |  | 39.29 | 5485.2 |  | 39.29 | 365.4 |
| 39.34 | 1219.4 |  | 39.34 | 4680.2 |  | 39.34 | 407.4 |
| 39.39 | 1190 |  | 39.39 | 3962 |  | 39.39 | 406 |
| 39.44 | 1188.6 |  | 39.44 | 3963.4 |  | 39.44 | 435.4 |
| 39.49 | 1092 |  | 39.49 | 3556 |  | 39.49 | 337.4 |
| 39.54 | 1062.6 |  | 39.54 | 3234 |  | 39.54 | 351.4 |
| 39.59 | 1136.8 |  | 39.59 | 3350.2 |  | 39.59 | 351.4 |
| 39.64 | 1136.8 |  | 39.64 | 3346 |  | 39.64 | 394.8 |
| 39.69 | 1275.4 |  | 39.69 | 3399.2 |  | 39.69 | 366.8 |
| 39.74 | 1442 |  | 39.74 | 3348.8 |  | 39.74 | 322 |
| 39.79 | 1516.2 |  | 39.79 | 3586.8 |  | 39.79 | 322 |
| 39.84 | 1513.4 |  | 39.84 | 3584 |  | 39.84 | 350 |
| 39.89 | 1513.4 |  | 39.89 | 3343.2 |  | 39.89 | 394.8 |
| 39.94 | 1579.2 |  | 39.94 | 3588.2 |  | 39.94 | 422.8 |
| 39.99 | 1332.8 |  | 39.99 | 3344.6 |  | 39.99 | 421.4 |
| 40.04 | 1330 |  | 40.04 | 3346 |  | 40.04 | 410.2 |
| 40.09 | 1244.6 |  | 40.09 | 3668 |  | 40.09 | 310.8 |
| 40.14 | 1064 |  | 40.14 | 3782.8 |  | 40.14 | 364 |
| 40.19 | 1120 |  | 40.19 | 4215.4 |  | 40.19 | 364 |
| 40.24 | 1122.8 |  | 40.24 | 4212.6 |  | 40.24 | 326.2 |
| 40.29 | 1248.8 |  | 40.29 | 4034.8 |  | 40.29 | 408.8 |
| 40.34 | 1318.8 |  | 40.34 | 3966.2 |  | 40.34 | 379.4 |
| 40.39 | 1484 |  | 40.39 | 3932.6 |  | 40.39 | 380.8 |
| 40.44 | 1482.6 |  | 40.44 | 3932.6 |  | 40.44 | 392 |
| 40.49 | 1523.2 |  | 40.49 | 3865.4 |  | 40.49 | 379.4 |
| 40.54 | 1625.4 |  | 40.54 | 3627.4 |  | 40.54 | 417.2 |
| 40.59 | 1988 |  | 40.59 | 3262 |  | 40.59 | 418.6 |
| 40.64 | 1990.8 |  | 40.64 | 3264.8 |  | 40.64 | 350 |
| 40.69 | 2074.8 |  | 40.69 | 3455.2 |  | 40.69 | 375.2 |
| 40.74 | 2087.4 |  | 40.74 | 3234 |  | 40.74 | 436.8 |
| 40.79 | 1794.8 |  | 40.79 | 3140.2 |  | 40.79 | 431.2 |
| 40.84 | 1792 |  | 40.84 | 3136 |  | 40.84 | 494.2 |
| 40.89 | 1411.2 |  | 40.89 | 3138.8 |  | 40.89 | 504 |
| 40.94 | 1230.6 |  | 40.94 | 3175.2 |  | 40.94 | 529.2 |
| 40.99 | 1023.4 |  | 40.99 | 3319.4 |  | 40.99 | 533.4 |
| 41.04 | 1020.6 |  | 41.04 | 3319.4 |  | 41.04 | 744.8 |
| 41.09 | 1078 |  | 41.09 | 3602.2 |  | 41.09 | 854 |
| 41.14 | 1134 |  | 41.14 | 3378.2 |  | 41.14 | 796.6 |
| 41.19 | 1191.4 |  | 41.19 | 3742.2 |  | 41.19 | 798 |
| 41.24 | 1188.6 |  | 41.24 | 3738 |  | 41.24 | 684.6 |
| 41.29 | 1232 |  | 41.29 | 3753.4 |  | 41.29 | 544.6 |
| 41.34 | 1453.2 |  | 41.34 | 4144 |  | 41.34 | 490 |
| 41.39 | 1624 |  | 41.39 | 5068 |  | 41.39 | 491.4 |
| 41.44 | 1624 |  | 41.44 | 5066.6 |  | 41.44 | 660.8 |
| 41.49 | 1820 |  | 41.49 | 5334 |  | 41.49 | 754.6 |
| 41.54 | 1887.2 |  | 41.54 | 5560.8 |  | 41.54 | 893.2 |
| 41.59 | 2214.8 |  | 41.59 | 4996.6 |  | 41.59 | 896 |
| 41.64 | 2214.8 |  | 41.64 | 5002.2 |  | 41.64 | 800.8 |
| 41.69 | 2424.8 |  | 41.69 | 3962 |  | 41.69 | 701.4 |
| 41.74 | 2800 |  | 41.74 | 3598 |  | 41.74 | 658 |
| 41.79 | 4016.6 |  | 41.79 | 3343.2 |  | 41.79 | 659.4 |
| 41.84 | 4016.6 |  | 41.84 | 3344.6 |  | 41.84 | 432.6 |
| 41.89 | 4718 |  | 41.89 | 3456.6 |  | 41.89 | 474.6 |
| 41.94 | 4608.8 |  | 41.94 | 3682 |  | 41.94 | 462 |
| 41.99 | 4128.6 |  | 41.99 | 3990 |  | 41.99 | 462 |
| 42.04 | 4130 |  | 42.04 | 3991.4 |  | 42.04 | 476 |
| 42.09 | 3386.6 |  | 42.09 | 4352.6 |  | 42.09 | 421.4 |
| 42.14 | 2494.8 |  | 42.14 | 4408.6 |  | 42.14 | 422.8 |
| 42.19 | 1789.2 |  | 42.19 | 4463.2 |  | 42.19 | 408.8 |
| 42.24 | 1790.6 |  | 42.24 | 4470.2 |  | 42.24 | 544.6 |
| 42.29 | 1429.4 |  | 42.29 | 4646.6 |  | 42.29 | 449.4 |
| 42.34 | 1454.6 |  | 42.34 | 4142.6 |  | 42.34 | 450.8 |
| 42.39 | 1320.2 |  | 42.39 | 3962 |  | 42.39 | 418.6 |
| 42.44 | 1316 |  | 42.44 | 3963.4 |  | 42.44 | 492.8 |
| 42.49 | 1286.6 |  | 42.49 | 4018 |  | 42.49 | 438.2 |
| 42.54 | 1289.4 |  | 42.54 | 4356.8 |  | 42.54 | 438.2 |
| 42.59 | 1159.2 |  | 42.59 | 4687.2 |  | 42.59 | 417.2 |
| 42.64 | 1164.8 |  | 42.64 | 4690 |  | 42.64 | 464.8 |
| 42.69 | 1191.4 |  | 42.69 | 5014.8 |  | 42.69 | 438.2 |
| 42.74 | 1162 |  | 42.74 | 4566.8 |  | 42.74 | 438.2 |
| 42.79 | 1135.4 |  | 42.79 | 4244.8 |  | 42.79 | 421.4 |
| 42.84 | 1132.6 |  | 42.84 | 4240.6 |  | 42.84 | 418.6 |
| 42.89 | 1173.2 |  | 42.89 | 3768.8 |  | 42.89 | 364 |
| 42.94 | 1246 |  | 42.94 | 3484.6 |  | 42.94 | 365.4 |
| 42.99 | 1328.6 |  | 42.99 | 3420.2 |  | 42.99 | 408.8 |
| 43.04 | 1331.4 |  | 43.04 | 3418.8 |  | 43.04 | 476 |
| 43.09 | 1568 |  | 43.09 | 3180.8 |  | 43.09 | 474.6 |
| 43.14 | 1894.2 |  | 43.14 | 3236.8 |  | 43.14 | 476 |
| 43.19 | 2517.2 |  | 43.19 | 2984.8 |  | 43.19 | 502.6 |
| 43.24 | 2520 |  | 43.24 | 2984.8 |  | 43.24 | 452.2 |
| 43.29 | 2898 |  | 43.29 | 3277.4 |  | 43.29 | 404.6 |
| 43.34 | 3262 |  | 43.34 | 3196.2 |  | 43.34 | 404.6 |
| 43.39 | 3483.2 |  | 43.39 | 3192 |  | 43.39 | 436.8 |
| 43.44 | 3486 |  | 43.44 | 3192 |  | 43.44 | 393.4 |
| 43.49 | 3250.8 |  | 43.49 | 3276 |  | 43.49 | 376.6 |
| 43.54 | 2675.4 |  | 43.54 | 3333.4 |  | 43.54 | 379.4 |
| 43.59 | 2517.2 |  | 43.59 | 3584 |  | 43.59 | 404.6 |
| 43.64 | 2524.2 |  | 43.64 | 3582.6 |  | 43.64 | 380.8 |
| 43.69 | 2672.6 |  | 43.69 | 3890.6 |  | 43.69 | 417.2 |
| 43.74 | 3066 |  | 43.74 | 4090.8 |  | 43.74 | 420 |
| 43.79 | 3516.8 |  | 43.79 | 4232.2 |  | 43.79 | 336 |
| 43.84 | 3515.4 |  | 43.84 | 4228 |  | 43.84 | 421.4 |
| 43.89 | 3544.8 |  | 43.89 | 4128.6 |  | 43.89 | 418.6 |
| 43.94 | 3249.4 |  | 43.94 | 4312 |  | 43.94 | 424.2 |
| 43.99 | 3220 |  | 43.99 | 4036.2 |  | 43.99 | 417.2 |
| 44.04 | 3220 |  | 44.04 | 4029.2 |  | 44.04 | 365.4 |
| 44.09 | 2734.2 |  | 44.09 | 3599.4 |  | 44.09 | 408.8 |
| 44.14 | 2170 |  | 44.14 | 3274.6 |  | 44.14 | 407.4 |
| 44.19 | 1836.8 |  | 44.19 | 3208.8 |  | 44.19 | 380.8 |
| 44.24 | 1835.4 |  | 44.24 | 3208.8 |  | 44.24 | 393.4 |
| 44.29 | 1804.6 |  | 44.29 | 3204.6 |  | 44.29 | 404.6 |
| 44.34 | 2002 |  | 44.34 | 3207.4 |  | 44.34 | 404.6 |
| 44.39 | 2870 |  | 44.39 | 3360 |  | 44.39 | 504 |
| 44.44 | 2868.6 |  | 44.44 | 3357.2 |  | 44.44 | 574 |
| 44.49 | 3920 |  | 44.49 | 3210.2 |  | 44.49 | 656.6 |
| 44.54 | 4855.2 |  | 44.54 | 3292.8 |  | 44.54 | 655.2 |
| 44.59 | 5054 |  | 44.59 | 3276 |  | 44.59 | 550.2 |
| 44.64 | 5056.8 |  | 44.64 | 3273.2 |  | 44.64 | 516.6 |
| 44.69 | 4593.4 |  | 44.69 | 3540.6 |  | 44.69 | 448 |
| 44.74 | 3694.6 |  | 44.74 | 3308.2 |  | 44.74 | 445.2 |
| 44.79 | 2846.2 |  | 44.79 | 3136 |  | 44.79 | 393.4 |
| 44.84 | 2843.4 |  | 44.84 | 3137.4 |  | 44.84 | 296.8 |
| 44.89 | 2633.4 |  | 44.89 | 3431.4 |  | 44.89 | 365.4 |
| 44.94 | 2314.2 |  | 44.94 | 3332 |  | 44.94 | 368.2 |
| 44.99 | 2324 |  | 44.99 | 3374 |  | 44.99 | 376.6 |
| 45.04 | 2326.8 |  | 45.04 | 3376.8 |  | 45.04 | 350 |
| 45.09 | 2296 |  | 45.09 | 3404.8 |  | 45.09 | 351.4 |
| 45.14 | 2450 |  | 45.14 | 3430 |  | 45.14 | 352.8 |
| 45.19 | 2634.8 |  | 45.19 | 3138.8 |  | 45.19 | 322 |
| 45.24 | 2630.6 |  | 45.24 | 3140.2 |  | 45.24 | 375.2 |
| 45.29 | 2662.8 |  | 45.29 | 3064.6 |  | 45.29 | 336 |
| 45.34 | 2532.6 |  | 45.34 | 3053.4 |  | 45.34 | 340.2 |
| 45.39 | 2241.4 |  | 45.39 | 2998.8 |  | 45.39 | 407.4 |
| 45.44 | 2241.4 |  | 45.44 | 2996 |  | 45.44 | 365.4 |
| 45.49 | 1817.2 |  | 45.49 | 3151.4 |  | 45.49 | 322 |
| 45.54 | 1551.2 |  | 45.54 | 3189.2 |  | 45.54 | 320.6 |
| 45.59 | 1470 |  | 45.59 | 3224.2 |  | 45.59 | 351.4 |
| 45.64 | 1471.4 |  | 45.64 | 3218.6 |  | 45.64 | 320.6 |
| 45.69 | 1597.4 |  | 45.69 | 3232.6 |  | 45.69 | 393.4 |
| 45.74 | 1555.4 |  | 45.74 | 3532.2 |  | 45.74 | 390.6 |
| 45.79 | 1892.8 |  | 45.79 | 3708.6 |  | 45.79 | 392 |
| 45.84 | 1887.2 |  | 45.84 | 3712.8 |  | 45.84 | 421.4 |
| 45.89 | 1976.8 |  | 45.89 | 4226.6 |  | 45.89 | 505.4 |
| 45.94 | 2002 |  | 45.94 | 4650.8 |  | 45.94 | 501.2 |
| 45.99 | 1724.8 |  | 45.99 | 5080.6 |  | 45.99 | 436.8 |
| 46.04 | 1726.2 |  | 46.04 | 5082 |  | 46.04 | 434 |
| 46.09 | 1398.6 |  | 46.09 | 4912.6 |  | 46.09 | 351.4 |
| 46.14 | 1334.2 |  | 46.14 | 4386.2 |  | 46.14 | 350 |
| 46.19 | 1271.2 |  | 46.19 | 4380.6 |  | 46.19 | 324.8 |
| 46.24 | 1275.4 |  | 46.24 | 4114.6 |  | 46.24 | 350 |
| 46.29 | 1412.6 |  | 46.29 | 3641.4 |  | 46.29 | 334.6 |
| 46.34 | 1608.6 |  | 46.34 | 3596.6 |  | 46.34 | 340.2 |
| 46.39 | 1737.4 |  | 46.39 | 3595.2 |  | 46.39 | 362.6 |
| 46.44 | 1734.6 |  | 46.44 | 3656.8 |  | 46.44 | 450.8 |
| 46.49 | 1850.8 |  | 46.49 | 3544.8 |  | 46.49 | 434 |
| 46.54 | 1653.4 |  | 46.54 | 3753.4 |  | 46.54 | 434 |
| 46.59 | 1454.6 |  | 46.59 | 3754.8 |  | 46.59 | 434 |
| 46.64 | 1458.8 |  | 46.64 | 3610.6 |  | 46.64 | 530.6 |
| 46.69 | 1244.6 |  | 46.69 | 3948 |  | 46.69 | 533.4 |
| 46.74 | 1348.2 |  | 46.74 | 4172 |  | 46.74 | 534.8 |
| 46.79 | 1327.2 |  | 46.79 | 4174.8 |  | 46.79 | 448 |
| 46.84 | 1328.6 |  | 46.84 | 4244.8 |  | 46.84 | 394.8 |
| 46.89 | 1205.4 |  | 46.89 | 4342.8 |  | 46.89 | 376.6 |
| 46.94 | 1260 |  | 46.94 | 4902.8 |  | 46.94 | 376.6 |
| 46.99 | 1290.8 |  | 46.99 | 4897.2 |  | 46.99 | 366.8 |
| 47.04 | 1290.8 |  | 47.04 | 5374.6 |  | 47.04 | 362.6 |
| 47.09 | 1470 |  | 47.09 | 5727.4 |  | 47.09 | 364 |
| 47.14 | 1614.2 |  | 47.14 | 5517.4 |  | 47.14 | 362.6 |
| 47.19 | 1684.2 |  | 47.19 | 5516 |  | 47.19 | 351.4 |
| 47.24 | 1678.6 |  | 47.24 | 5248.6 |  | 47.24 | 310.8 |
| 47.29 | 1600.2 |  | 47.29 | 4340 |  | 47.29 | 319.2 |
| 47.34 | 1638 |  | 47.34 | 3560.2 |  | 47.34 | 324.8 |
| 47.39 | 1640.8 |  | 47.39 | 3556 |  | 47.39 | 404.6 |
| 47.44 | 1705.2 |  | 47.44 | 3346 |  | 47.44 | 376.6 |
| 47.49 | 2168.6 |  | 47.49 | 3136 |  | 47.49 | 435.4 |
| 47.54 | 2592.8 |  | 47.54 | 2970.8 |  | 47.54 | 431.2 |
| 47.59 | 2591.4 |  | 47.59 | 2970.8 |  | 47.59 | 396.2 |
| 47.64 | 2676.8 |  | 47.64 | 3336.2 |  | 47.64 | 382.2 |
| 47.69 | 2534 |  | 47.69 | 3175.2 |  | 47.69 | 448 |
| 47.74 | 2521.4 |  | 47.74 | 3094 |  | 47.74 | 449.4 |
| 47.79 | 2521.4 |  | 47.79 | 3095.4 |  | 47.79 | 445.2 |
| 47.84 | 2406.6 |  | 47.84 | 3290 |  | 47.84 | 407.4 |
| 47.89 | 2244.2 |  | 47.89 | 3386.6 |  | 47.89 | 406 |
| 47.94 | 2014.6 |  | 47.94 | 3238.2 |  | 47.94 | 407.4 |
| 47.99 | 2017.4 |  | 47.99 | 3235.4 |  | 47.99 | 323.4 |
| 48.04 | 1723.4 |  | 48.04 | 3138.8 |  | 48.04 | 365.4 |
| 48.09 | 1429.4 |  | 48.09 | 3264.8 |  | 48.09 | 338.8 |
| 48.14 | 1164.8 |  | 48.14 | 3502.8 |  | 48.14 | 333.2 |
| 48.19 | 1166.2 |  | 48.19 | 3504.2 |  | 48.19 | 348.6 |
| 48.24 | 1050 |  | 48.24 | 3428.6 |  | 48.24 | 323.4 |
| 48.29 | 1009.4 |  | 48.29 | 3473.4 |  | 48.29 | 338.8 |
| 48.34 | 981.4 |  | 48.34 | 3526.6 |  | 48.34 | 333.2 |
| 48.39 | 980 |  | 48.39 | 3530.8 |  | 48.39 | 436.8 |
| 48.44 | 939.4 |  | 48.44 | 3375.4 |  | 48.44 | 348.6 |
| 48.49 | 1038.8 |  | 48.49 | 3304 |  | 48.49 | 322 |
| 48.54 | 1076.6 |  | 48.54 | 3264.8 |  | 48.54 | 322 |
| 48.59 | 1082.2 |  | 48.59 | 3264.8 |  | 48.59 | 336 |
| 48.64 | 1026.2 |  | 48.64 | 3140.2 |  | 48.64 | 323.4 |
| 48.69 | 1037.4 |  | 48.69 | 3176.6 |  | 48.69 | 365.4 |
| 48.74 | 1079.4 |  | 48.74 | 3249.4 |  | 48.74 | 368.2 |
| 48.79 | 1078 |  | 48.79 | 3250.8 |  | 48.79 | 379.4 |
| 48.84 | 978.6 |  | 48.84 | 3189.2 |  | 48.84 | 350 |
| 48.89 | 994 |  | 48.89 | 3218.6 |  | 48.89 | 338.8 |
| 48.94 | 984.2 |  | 48.94 | 3236.8 |  | 48.94 | 337.4 |
| 48.99 | 980 |  | 48.99 | 3234 |  | 48.99 | 366.8 |
| 49.04 | 1010.8 |  | 49.04 | 3138.8 |  | 49.04 | 350 |
| 49.09 | 940.8 |  | 49.09 | 3164 |  | 49.09 | 322 |
| 49.14 | 883.4 |  | 49.14 | 3222.8 |  | 49.14 | 326.2 |
| 49.19 | 884.8 |  | 49.19 | 3222.8 |  | 49.19 | 305.2 |
| 49.24 | 924 |  | 49.24 | 3077.2 |  | 49.24 | 365.4 |
| 49.29 | 1066.8 |  | 49.29 | 3231.2 |  | 49.29 | 306.6 |
| 49.34 | 950.6 |  | 49.34 | 3238.2 |  | 49.34 | 312.2 |
| 49.39 | 950.6 |  | 49.39 | 3232.6 |  | 49.39 | 336 |
| 49.44 | 1006.6 |  | 49.44 | 3092.6 |  | 49.44 | 336 |
| 49.49 | 908.6 |  | 49.49 | 3064.6 |  | 49.49 | 350 |
| 49.54 | 1024.8 |  | 49.54 | 3039.4 |  | 49.54 | 351.4 |
| 49.59 | 1023.4 |  | 49.59 | 3038 |  | 49.59 | 320.6 |
| 49.64 | 994 |  | 49.64 | 2980.6 |  | 49.64 | 294 |
| 49.69 | 1079.4 |  | 49.69 | 2997.4 |  | 49.69 | 326.2 |
| 49.74 | 1064 |  | 49.74 | 3147.2 |  | 49.74 | 320.6 |
| 49.79 | 1068.2 |  | 49.79 | 3151.4 |  | 49.79 | 294 |
| 49.84 | 1064 |  | 49.84 | 3264.8 |  | 49.84 | 310.8 |
| 49.89 | 1118.6 |  | 49.89 | 3161.2 |  | 49.89 | 337.4 |
| 49.94 | 1050 |  | 49.94 | 3165.4 |  | 49.94 | 336 |
| 49.99 | 1050 |  | 49.99 | 3164 |  | 49.99 | 378 |
| 50.04 | 1052.8 |  | 50.04 | 3222.8 |  | 50.04 | 308 |
| 50.09 | 1120 |  | 50.09 | 3278.8 |  | 50.09 | 380.8 |
| 50.14 | 1190 |  | 50.14 | 3574.2 |  | 50.14 | 379.4 |
| 50.19 | 1194.2 |  | 50.19 | 3567.2 |  | 50.19 | 380.8 |
| 50.24 | 1176 |  | 50.24 | 3850 |  | 50.24 | 352.8 |
| 50.29 | 1276.8 |  | 50.29 | 3949.4 |  | 50.29 | 336 |
| 50.34 | 1593.2 |  | 50.34 | 3894.8 |  | 50.34 | 334.6 |
| 50.39 | 1597.4 |  | 50.39 | 3890.6 |  | 50.39 | 277.2 |
| 50.44 | 1878.8 |  | 50.44 | 3838.8 |  | 50.44 | 326.2 |
| 50.49 | 2254 |  | 50.49 | 3586.8 |  | 50.49 | 324.8 |
| 50.54 | 2490.6 |  | 50.54 | 3260.6 |  | 50.54 | 296.8 |
| 50.59 | 2494.8 |  | 50.59 | 3264.8 |  | 50.59 | 268.8 |
| 50.64 | 2489.2 |  | 50.64 | 3235.4 |  | 50.64 | 376.6 |
| 50.69 | 2433.2 |  | 50.69 | 3288.6 |  | 50.69 | 379.4 |
| 50.74 | 2552.2 |  | 50.74 | 3301.2 |  | 50.74 | 337.4 |
| 50.79 | 2549.4 |  | 50.79 | 3302.6 |  | 50.79 | 264.6 |
| 50.84 | 2842 |  | 50.84 | 3316.6 |  | 50.84 | 280 |
| 50.89 | 2784.6 |  | 50.89 | 3539.2 |  | 50.89 | 282.8 |
| 50.94 | 2856 |  | 50.94 | 3414.6 |  | 50.94 | 266 |
| 50.99 | 2854.6 |  | 50.99 | 3418.8 |  | 50.99 | 282.8 |
| 51.04 | 2451.4 |  | 51.04 | 3193.4 |  | 51.04 | 323.4 |
| 51.09 | 2000.6 |  | 51.09 | 3346 |  | 51.09 | 322 |
| 51.14 | 1527.4 |  | 51.14 | 3330.6 |  | 51.14 | 296.8 |
| 51.19 | 1526 |  | 51.19 | 3334.8 |  | 51.19 | 361.2 |
| 51.24 | 1376.2 |  | 51.24 | 3301.2 |  | 51.24 | 348.6 |
| 51.29 | 1400 |  | 51.29 | 3306.8 |  | 51.29 | 350 |
| 51.34 | 1499.4 |  | 51.34 | 3320.8 |  | 51.34 | 382.2 |
| 51.39 | 1496.6 |  | 51.39 | 3315.2 |  | 51.39 | 376.6 |
| 51.44 | 1513.4 |  | 51.44 | 3189.2 |  | 51.44 | 376.6 |
| 51.49 | 1496.6 |  | 51.49 | 2830.8 |  | 51.49 | 379.4 |
| 51.54 | 1332.8 |  | 51.54 | 3081.4 |  | 51.54 | 337.4 |
| 51.59 | 1328.6 |  | 51.59 | 3080 |  | 51.59 | 324.8 |
| 51.64 | 1285.2 |  | 51.64 | 2942.8 |  | 51.64 | 267.4 |
| 51.69 | 1345.4 |  | 51.69 | 2854.6 |  | 51.69 | 270.2 |
| 51.74 | 1640.8 |  | 51.74 | 2826.6 |  | 51.74 | 310.8 |
| 51.79 | 1635.2 |  | 51.79 | 2828 |  | 51.79 | 268.8 |
| 51.84 | 1906.8 |  | 51.84 | 2940 |  | 51.84 | 278.6 |
| 51.89 | 2451.4 |  | 51.89 | 2927.4 |  | 51.89 | 281.4 |
| 51.94 | 2629.2 |  | 51.94 | 2870 |  | 51.94 | 296.8 |
| 51.99 | 2634.8 |  | 51.99 | 2872.8 |  | 51.99 | 322 |
| 52.04 | 2618 |  | 52.04 | 2662.8 |  | 52.04 | 291.2 |
| 52.09 | 2517.2 |  | 52.09 | 2846.2 |  | 52.09 | 292.6 |
| 52.14 | 2298.8 |  | 52.14 | 2983.4 |  | 52.14 | 333.2 |
| 52.19 | 2296 |  | 52.19 | 2980.6 |  | 52.19 | 278.6 |
| 52.24 | 2170 |  | 52.24 | 3196.2 |  | 52.24 | 263.2 |
| 52.29 | 1824.2 |  | 52.29 | 3277.4 |  | 52.29 | 270.2 |
| 52.34 | 1600.2 |  | 52.34 | 3330.6 |  | 52.34 | 351.4 |
| 52.39 | 1594.6 |  | 52.39 | 3332 |  | 52.39 | 322 |
| 52.44 | 1596 |  | 52.44 | 3066 |  | 52.44 | 296.8 |
| 52.49 | 1596 |  | 52.49 | 3011.4 |  | 52.49 | 291.2 |
| 52.54 | 1779.4 |  | 52.54 | 3165.4 |  | 52.54 | 324.8 |
| 52.59 | 1779.4 |  | 52.59 | 3164 |  | 52.59 | 376.6 |
| 52.64 | 1611.4 |  | 52.64 | 3022.6 |  | 52.64 | 305.2 |
| 52.69 | 1790.6 |  | 52.69 | 2910.6 |  | 52.69 | 312.2 |
| 52.74 | 1972.6 |  | 52.74 | 2882.6 |  | 52.74 | 278.6 |
| 52.79 | 1978.2 |  | 52.79 | 2884 |  | 52.79 | 308 |
| 52.84 | 2171.4 |  | 52.84 | 2746.8 |  | 52.84 | 292.6 |
| 52.89 | 2172.8 |  | 52.89 | 3011.4 |  | 52.89 | 291.2 |
| 52.94 | 2184 |  | 52.94 | 2857.4 |  | 52.94 | 312.2 |
| 52.99 | 2185.4 |  | 52.99 | 2856 |  | 52.99 | 352.8 |
| 53.04 | 2438.8 |  | 53.04 | 3064.6 |  | 53.04 | 295.4 |
| 53.09 | 2256.8 |  | 53.09 | 2910.6 |  | 53.09 | 296.8 |
| 53.14 | 2240 |  | 53.14 | 2886.8 |  | 53.14 | 291.2 |
| 53.19 | 2240 |  | 53.19 | 2884 |  | 53.19 | 250.6 |
| 53.24 | 2157.4 |  | 53.24 | 2900.8 |  | 53.24 | 295.4 |
| 53.29 | 1888.6 |  | 53.29 | 2938.6 |  | 53.29 | 294 |
| 53.34 | 1877.4 |  | 53.34 | 2926 |  | 53.34 | 292.6 |
| 53.39 | 1874.6 |  | 53.39 | 2928.8 |  | 53.39 | 263.2 |
| 53.44 | 1920.8 |  | 53.44 | 2996 |  | 53.44 | 306.6 |
| 53.49 | 1834 |  | 53.49 | 3064.6 |  | 53.49 | 310.8 |
| 53.54 | 1737.4 |  | 53.54 | 2984.8 |  | 53.54 | 242.2 |
| 53.59 | 1737.4 |  | 53.59 | 2982 |  | 53.59 | 296.8 |
| 53.64 | 1593.2 |  | 53.64 | 2755.2 |  | 53.64 | 292.6 |
| 53.69 | 1374.8 |  | 53.69 | 2756.6 |  | 53.69 | 291.2 |
| 53.74 | 1146.6 |  | 53.74 | 2774.8 |  | 53.74 | 291.2 |
| 53.79 | 1145.2 |  | 53.79 | 2773.4 |  | 53.79 | 338.8 |
| 53.84 | 1162 |  | 53.84 | 2812.6 |  | 53.84 | 323.4 |
| 53.89 | 1150.8 |  | 53.89 | 2672.6 |  | 53.89 | 322 |
| 53.94 | 1103.2 |  | 53.94 | 2898 |  | 53.94 | 320.6 |
| 53.99 | 1106 |  | 53.99 | 2898 |  | 53.99 | 281.4 |
| 54.04 | 1160.6 |  | 54.04 | 2982 |  | 54.04 | 249.2 |
| 54.09 | 1304.8 |  | 54.09 | 2958.2 |  | 54.09 | 254.8 |
| 54.14 | 1418.2 |  | 54.14 | 2854.6 |  | 54.14 | 266 |
| 54.19 | 1418.2 |  | 54.19 | 2860.2 |  | 54.19 | 309.4 |
| 54.24 | 1303.4 |  | 54.24 | 2926 |  | 54.24 | 240.8 |
| 54.29 | 1289.4 |  | 54.29 | 2956.8 |  | 54.29 | 239.4 |
| 54.34 | 1384.6 |  | 54.34 | 3025.4 |  | 54.34 | 309.4 |
| 54.39 | 1390.2 |  | 54.39 | 3024 |  | 54.39 | 282.8 |
| 54.44 | 1376.2 |  | 54.44 | 2986.2 |  | 54.44 | 310.8 |
| 54.49 | 1402.8 |  | 54.49 | 2881.2 |  | 54.49 | 306.6 |
| 54.54 | 1486.8 |  | 54.54 | 2858.8 |  | 54.54 | 270.2 |
| 54.59 | 1481.2 |  | 54.59 | 2857.4 |  | 54.59 | 239.4 |
| 54.64 | 1500.8 |  | 54.64 | 2895.2 |  | 54.64 | 281.4 |
| 54.69 | 1390.2 |  | 54.69 | 3022.6 |  | 54.69 | 281.4 |
| 54.74 | 1488.2 |  | 54.74 | 3022.6 |  | 54.74 | 253.4 |
| 54.79 | 1486.8 |  | 54.79 | 2846.2 |  | 54.79 | 256.2 |
| 54.84 | 1537.2 |  | 54.84 | 2814 |  | 54.84 | 267.4 |
| 54.89 | 1524.6 |  | 54.89 | 2954 |  | 54.89 | 270.2 |
| 54.94 | 1509.2 |  | 54.94 | 2954 |  | 54.94 | 256.2 |
| 54.99 | 1513.4 |  | 54.99 | 3095.4 |  | 54.99 | 277.2 |
| 55.04 | 1218 |  | 55.04 | 3120.6 |  | 55.04 | 254.8 |
| 55.09 | 1178.8 |  | 55.09 | 3348.8 |  | 55.09 | 253.4 |
| 55.14 | 1026.2 |  | 55.14 | 3346 |  | 55.14 | 278.6 |
| 55.19 | 1020.6 |  | 55.19 | 3263.4 |  | 55.19 | 282.8 |
| 55.24 | 1066.8 |  | 55.24 | 3136 |  | 55.24 | 294 |
| 55.29 | 1064 |  | 55.29 | 3147.2 |  | 55.29 | 296.8 |
| 55.34 | 1068.2 |  | 55.34 | 3152.8 |  | 55.34 | 264.6 |
| 55.39 | 1065.4 |  | 55.39 | 3066 |  | 55.39 | 235.2 |
| 55.44 | 1122.8 |  | 55.44 | 2910.6 |  | 55.44 | 291.2 |
| 55.49 | 1050 |  | 55.49 | 2884 |  | 55.49 | 296.8 |
| 55.54 | 1162 |  | 55.54 | 2882.6 |  | 55.54 | 364 |
| 55.59 | 1162 |  | 55.59 | 2952.6 |  | 55.59 | 278.6 |
| 55.64 | 1166.2 |  | 55.64 | 2912 |  | 55.64 | 253.4 |
| 55.69 | 1288 |  | 55.69 | 2969.4 |  | 55.69 | 254.8 |
| 55.74 | 1288 |  | 55.74 | 2972.2 |  | 55.74 | 267.4 |
| 55.79 | 1442 |  | 55.79 | 3190.6 |  | 55.79 | 323.4 |
| 55.84 | 1537.2 |  | 55.84 | 2956.8 |  | 55.84 | 310.8 |
| 55.89 | 1625.4 |  | 55.89 | 2954 |  | 55.89 | 308 |
| 55.94 | 1625.4 |  | 55.94 | 2951.2 |  | 55.94 | 338.8 |
| 55.99 | 1607.2 |  | 55.99 | 2968 |  | 55.99 | 362.6 |
| 56.04 | 1642.2 |  | 56.04 | 3050.6 |  | 56.04 | 348.6 |
| 56.09 | 1612.8 |  | 56.09 | 3238.2 |  | 56.09 | 352.8 |
| 56.14 | 1608.6 |  | 56.14 | 3231.2 |  | 56.14 | 264.6 |
| 56.19 | 1302 |  | 56.19 | 3208.8 |  | 56.19 | 334.6 |
| 56.24 | 1261.4 |  | 56.24 | 3484.6 |  | 56.24 | 350 |
| 56.29 | 1388.8 |  | 56.29 | 3220 |  | 56.29 | 351.4 |
| 56.34 | 1388.8 |  | 56.34 | 3220 |  | 56.34 | 350 |
| 56.39 | 1289.4 |  | 56.39 | 3290 |  | 56.39 | 350 |
| 56.44 | 1372 |  | 56.44 | 3108 |  | 56.44 | 295.4 |
| 56.49 | 1726.2 |  | 56.49 | 3122 |  | 56.49 | 298.2 |
| 56.54 | 1724.8 |  | 56.54 | 3123.4 |  | 56.54 | 308 |
| 56.59 | 1750 |  | 56.59 | 3333.4 |  | 56.59 | 361.2 |
| 56.64 | 1737.4 |  | 56.64 | 3248 |  | 56.64 | 323.4 |
| 56.69 | 1640.8 |  | 56.69 | 3560.2 |  | 56.69 | 324.8 |
| 56.74 | 1639.4 |  | 56.74 | 3553.2 |  | 56.74 | 291.2 |
| 56.79 | 1344 |  | 56.79 | 3504.2 |  | 56.79 | 295.4 |
| 56.84 | 1206.8 |  | 56.84 | 3680.6 |  | 56.84 | 250.6 |
| 56.89 | 1202.6 |  | 56.89 | 3194.8 |  | 56.89 | 250.6 |
| 56.94 | 1204 |  | 56.94 | 3196.2 |  | 56.94 | 348.6 |
| 56.99 | 1236.2 |  | 56.99 | 3266.2 |  | 56.99 | 253.4 |
| 57.04 | 1092 |  | 57.04 | 3067.4 |  | 57.04 | 295.4 |
| 57.09 | 1149.4 |  | 57.09 | 3137.4 |  | 57.09 | 294 |
| 57.14 | 1152.2 |  | 57.14 | 3134.6 |  | 57.14 | 322 |
| 57.19 | 1149.4 |  | 57.19 | 3081.4 |  | 57.19 | 280 |
| 57.24 | 1138.2 |  | 57.24 | 2884 |  | 57.24 | 294 |
| 57.29 | 1096.2 |  | 57.29 | 2941.4 |  | 57.29 | 294 |
| 57.34 | 1094.8 |  | 57.34 | 2938.6 |  | 57.34 | 352.8 |
| 57.39 | 1107.4 |  | 57.39 | 2882.6 |  | 57.39 | 294 |
| 57.44 | 1192.8 |  | 57.44 | 2885.4 |  | 57.44 | 264.6 |
| 57.49 | 1160.6 |  | 57.49 | 2968 |  | 57.49 | 263.2 |
| 57.54 | 1160.6 |  | 57.54 | 2968 |  | 57.54 | 284.2 |
| 57.59 | 1246 |  | 57.59 | 2968 |  | 57.59 | 222.6 |
| 57.64 | 1373.4 |  | 57.64 | 2856 |  | 57.64 | 252 |
| 57.69 | 1146.6 |  | 57.69 | 2900.8 |  | 57.69 | 253.4 |
| 57.74 | 1146.6 |  | 57.74 | 2899.4 |  | 57.74 | 280 |
| 57.79 | 1134 |  | 57.79 | 2853.2 |  | 57.79 | 281.4 |
| 57.84 | 1122.8 |  | 57.84 | 2940 |  | 57.84 | 266 |
| 57.89 | 1163.4 |  | 57.89 | 2787.4 |  | 57.89 | 267.4 |
| 57.94 | 1164.8 |  | 57.94 | 2784.6 |  | 57.94 | 310.8 |
| 57.99 | 1246 |  | 57.99 | 3050.6 |  | 57.99 | 336 |
| 58.04 | 1250.2 |  | 58.04 | 2969.4 |  | 58.04 | 281.4 |
| 58.09 | 1412.6 |  | 58.09 | 3133.2 |  | 58.09 | 284.2 |
| 58.14 | 1416.8 |  | 58.14 | 3133.2 |  | 58.14 | 351.4 |
| 58.19 | 1836.8 |  | 58.19 | 3040.8 |  | 58.19 | 252 |
| 58.24 | 1778 |  | 58.24 | 2774.8 |  | 58.24 | 252 |
| 58.29 | 1720.6 |  | 58.29 | 2885.4 |  | 58.29 | 309.4 |
| 58.34 | 1720.6 |  | 58.34 | 2886.8 |  | 58.34 | 306.6 |
| 58.39 | 1666 |  | 58.39 | 2703.4 |  | 58.39 | 319.2 |
| 58.44 | 1474.2 |  | 58.44 | 2619.4 |  | 58.44 | 326.2 |
| 58.49 | 1470 |  | 58.49 | 2660 |  | 58.49 | 322 |
| 58.54 | 1468.6 |  | 58.54 | 2662.8 |  | 58.54 | 338.8 |
| 58.59 | 1314.6 |  | 58.59 | 2674 |  | 58.59 | 336 |
| 58.64 | 1302 |  | 58.64 | 3010 |  | 58.64 | 296.8 |
| 58.69 | 1230.6 |  | 58.69 | 2871.4 |  | 58.69 | 296.8 |
| 58.74 | 1233.4 |  | 58.74 | 2871.4 |  | 58.74 | 324.8 |
| 58.79 | 1386 |  | 58.79 | 2700.6 |  | 58.79 | 312.2 |
| 58.84 | 1523.2 |  | 58.84 | 2690.8 |  | 58.84 | 308 |
| 58.89 | 1775.2 |  | 58.89 | 2944.2 |  | 58.89 | 309.4 |
| 58.94 | 1782.2 |  | 58.94 | 2940 |  | 58.94 | 278.6 |
| 58.99 | 1989.4 |  | 58.99 | 2776.2 |  | 58.99 | 348.6 |
| 59.04 | 2112.6 |  | 59.04 | 2858.8 |  | 59.04 | 352.8 |
| 59.09 | 2328.2 |  | 59.09 | 2886.8 |  | 59.09 | 334.6 |
| 59.14 | 2325.4 |  | 59.14 | 2886.8 |  | 59.14 | 366.8 |
| 59.19 | 2396.8 |  | 59.19 | 2825.2 |  | 59.19 | 338.8 |
| 59.24 | 2410.8 |  | 59.24 | 2842 |  | 59.24 | 333.2 |
| 59.29 | 2464 |  | 59.29 | 2828 |  | 59.29 | 324.8 |
| 59.34 | 2464 |  | 59.34 | 2832.2 |  | 59.34 | 326.2 |
| 59.39 | 2115.4 |  | 59.39 | 2951.2 |  | 59.39 | 277.2 |
| 59.44 | 1708 |  | 59.44 | 2756.6 |  | 59.44 | 280 |
| 59.49 | 1527.4 |  | 59.49 | 2970.8 |  | 59.49 | 208.6 |
| 59.54 | 1523.2 |  | 59.54 | 2968 |  | 59.54 | 305.2 |
| 59.59 | 1416.8 |  | 59.59 | 3038 |  | 59.59 | 250.6 |
| 59.64 | 1149.4 |  | 59.64 | 2956.8 |  | 59.64 | 253.4 |
| 59.69 | 1219.4 |  | 59.69 | 3039.4 |  | 59.69 | 282.8 |
| 59.74 | 1220.8 |  | 59.74 | 3039.4 |  | 59.74 | 309.4 |
| 59.79 | 1121.4 |  | 59.79 | 3290 |  | 59.79 | 324.8 |
| 59.84 | 1290.8 |  | 59.84 | 3501.4 |  | 59.84 | 319.2 |
| 59.89 | 1300.6 |  | 59.89 | 3599.4 |  | 59.89 | 308 |
| 59.94 | 1306.2 |  | 59.94 | 3599.4 |  | 59.94 | 295.4 |
| 59.99 | 1187.2 |  | 59.99 | 3498.6 |  | 59.99 | 350 |
| 60.04 | 1328.6 |  | 60.04 | 3641.4 |  | 60.04 | 348.6 |
| 60.09 | 1177.4 |  | 60.09 | 3406.2 |  | 60.09 | 308 |
| 60.14 | 1174.6 |  | 60.14 | 3402 |  | 60.14 | 277.2 |
| 60.19 | 1247.4 |  | 60.19 | 3414.6 |  | 60.19 | 253.4 |
| 60.24 | 1131.2 |  | 60.24 | 3404.8 |  | 60.24 | 253.4 |
| 60.29 | 1230.6 |  | 60.29 | 3474.8 |  | 60.29 | 337.4 |
| 60.34 | 1232 |  | 60.34 | 3469.2 |  | 60.34 | 334.6 |
| 60.39 | 1229.2 |  | 60.39 | 3574.2 |  | 60.39 | 326.2 |
| 60.44 | 1432.2 |  | 60.44 | 3764.6 |  | 60.44 | 319.2 |
| 60.49 | 1446.2 |  | 60.49 | 3568.6 |  | 60.49 | 338.8 |
| 60.54 | 1446.2 |  | 60.54 | 3570 |  | 60.54 | 420 |
| 60.59 | 1524.6 |  | 60.59 | 3750.6 |  | 60.59 | 352.8 |
| 60.64 | 1586.2 |  | 60.64 | 3406.2 |  | 60.64 | 352.8 |
| 60.69 | 1593.2 |  | 60.69 | 3371.2 |  | 60.69 | 379.4 |
| 60.74 | 1596 |  | 60.74 | 3371.2 |  | 60.74 | 306.6 |
| 60.79 | 1572.2 |  | 60.79 | 3371.2 |  | 60.79 | 368.2 |
| 60.84 | 1582 |  | 60.84 | 3315.2 |  | 60.84 | 361.2 |
| 60.89 | 1530.2 |  | 60.89 | 3432.8 |  | 60.89 | 351.4 |
| 60.94 | 1526 |  | 60.94 | 3430 |  | 60.94 | 337.4 |
| 60.99 | 1471.4 |  | 60.99 | 3098.2 |  | 60.99 | 296.8 |
| 61.04 | 1316 |  | 61.04 | 3054.8 |  | 61.04 | 298.2 |
| 61.09 | 1247.4 |  | 61.09 | 3025.4 |  | 61.09 | 291.2 |
| 61.14 | 1246 |  | 61.14 | 3025.4 |  | 61.14 | 306.6 |
| 61.19 | 1121.4 |  | 61.19 | 2954 |  | 61.19 | 308 |
| 61.24 | 1066.8 |  | 61.24 | 2811.2 |  | 61.24 | 310.8 |
| 61.29 | 1010.8 |  | 61.29 | 2839.2 |  | 61.29 | 296.8 |
| 61.34 | 1009.4 |  | 61.34 | 2840.6 |  | 61.34 | 267.4 |
| 61.39 | 926.8 |  | 61.39 | 3000.2 |  | 61.39 | 294 |
| 61.44 | 952 |  | 61.44 | 2954 |  | 61.44 | 291.2 |
| 61.49 | 935.2 |  | 61.49 | 2856 |  | 61.49 | 295.4 |
| 61.54 | 936.6 |  | 61.54 | 2858.8 |  | 61.54 | 324.8 |
| 61.59 | 922.6 |  | 61.59 | 2914.8 |  | 61.59 | 282.8 |
| 61.64 | 852.6 |  | 61.64 | 2968 |  | 61.64 | 280 |
| 61.69 | 1080.8 |  | 61.69 | 3008.6 |  | 61.69 | 323.4 |
| 61.74 | 1078 |  | 61.74 | 3011.4 |  | 61.74 | 323.4 |
| 61.79 | 1090.6 |  | 61.79 | 2944.2 |  | 61.79 | 264.6 |
| 61.84 | 1244.6 |  | 61.84 | 2940 |  | 61.84 | 266 |
| 61.89 | 1117.2 |  | 61.89 | 2910.6 |  | 61.89 | 378 |
| 61.94 | 1118.6 |  | 61.94 | 2913.4 |  | 61.94 | 407.4 |
| 61.99 | 1334.2 |  | 61.99 | 3148.6 |  | 61.99 | 365.4 |
| 62.04 | 1316 |  | 62.04 | 3109.4 |  | 62.04 | 368.2 |
| 62.09 | 1264.2 |  | 62.09 | 3178 |  | 62.09 | 432.6 |
| 62.14 | 1258.6 |  | 62.14 | 3179.4 |  | 62.14 | 393.4 |
| 62.19 | 1264.2 |  | 62.19 | 2984.8 |  | 62.19 | 382.2 |
| 62.24 | 1047.2 |  | 62.24 | 3147.2 |  | 62.24 | 379.4 |
| 62.29 | 966 |  | 62.29 | 2924.6 |  | 62.29 | 389.2 |
| 62.34 | 966 |  | 62.34 | 2930.2 |  | 62.34 | 324.8 |
| 62.39 | 1076.6 |  | 62.39 | 2966.6 |  | 62.39 | 352.8 |
| 62.44 | 1012.2 |  | 62.44 | 2867.2 |  | 62.44 | 348.6 |
| 62.49 | 1008 |  | 62.49 | 2830.8 |  | 62.49 | 296.8 |
| 62.54 | 1005.2 |  | 62.54 | 2828 |  | 62.54 | 306.6 |
| 62.59 | 1230.6 |  | 62.59 | 2870 |  | 62.59 | 322 |
| 62.64 | 1190 |  | 62.64 | 2899.4 |  | 62.64 | 324.8 |
| 62.69 | 1362.2 |  | 62.69 | 2830.8 |  | 62.69 | 292.6 |
| 62.74 | 1360.8 |  | 62.74 | 2830.8 |  | 62.74 | 319.2 |
| 62.79 | 1334.2 |  | 62.79 | 3008.6 |  | 62.79 | 320.6 |
| 62.84 | 1486.8 |  | 62.84 | 2858.8 |  | 62.84 | 323.4 |
| 62.89 | 1527.4 |  | 62.89 | 2758 |  | 62.89 | 366.8 |
| 62.94 | 1524.6 |  | 62.94 | 2756.6 |  | 62.94 | 347.2 |
| 62.99 | 1608.6 |  | 62.99 | 2716 |  | 62.99 | 312.2 |
| 63.04 | 1523.2 |  | 63.04 | 2872.8 |  | 63.04 | 308 |
| 63.09 | 1488.2 |  | 63.09 | 2870 |  | 63.09 | 280 |
| 63.14 | 1486.8 |  | 63.14 | 2830.8 |  | 63.14 | 282.8 |
| 63.19 | 1374.8 |  | 63.19 | 2914.8 |  | 63.19 | 291.2 |
| 63.24 | 1303.4 |  | 63.24 | 2926 |  | 63.24 | 291.2 |
| 63.29 | 1244.6 |  | 63.29 | 2924.6 |  | 63.29 | 310.8 |
| 63.34 | 1244.6 |  | 63.34 | 2942.8 |  | 63.34 | 210 |
| 63.39 | 1037.4 |  | 63.39 | 3007.2 |  | 63.39 | 266 |
| 63.44 | 1047.2 |  | 63.44 | 3064.6 |  | 63.44 | 267.4 |
| 63.49 | 1066.8 |  | 63.49 | 3070.2 |  | 63.49 | 284.2 |
| 63.54 | 1064 |  | 63.54 | 3092.6 |  | 63.54 | 305.2 |
| 63.59 | 994 |  | 63.59 | 3165.4 |  | 63.59 | 239.4 |
| 63.64 | 1020.6 |  | 63.64 | 3178 |  | 63.64 | 238 |
| 63.69 | 1160.6 |  | 63.69 | 3180.8 |  | 63.69 | 254.8 |
| 63.74 | 1166.2 |  | 63.74 | 3011.4 |  | 63.74 | 266 |
| 63.79 | 1220.8 |  | 63.79 | 2856 |  | 63.79 | 211.4 |
| 63.84 | 1062.6 |  | 63.84 | 2675.4 |  | 63.84 | 208.6 |
| 63.89 | 1108.8 |  | 63.89 | 2675.4 |  | 63.89 | 280 |
| 63.94 | 1108.8 |  | 63.94 | 2790.2 |  | 63.94 | 238 |
| 63.99 | 1050 |  | 63.99 | 2853.2 |  | 63.99 | 253.4 |
| 64.04 | 1120 |  | 64.04 | 2854.6 |  | 64.04 | 250.6 |
| 64.09 | 1122.8 |  | 64.09 | 2857.4 |  | 64.09 | 254.8 |
| 64.14 | 1009.4 |  | 64.14 | 2758 |  | 64.14 | 253.4 |
| 64.19 | 1037.4 |  | 64.19 | 2914.8 |  | 64.19 | 221.2 |
| 64.24 | 1162 |  | 64.24 | 2839.2 |  | 64.24 | 222.6 |
| 64.29 | 1162 |  | 64.29 | 2843.4 |  | 64.29 | 291.2 |
| 64.34 | 1110.2 |  | 64.34 | 2828 |  | 64.34 | 306.6 |
| 64.39 | 1054.2 |  | 64.39 | 2760.8 |  | 64.39 | 284.2 |
| 64.44 | 1078 |  | 64.44 | 2826.6 |  | 64.44 | 280 |
| 64.49 | 1078 |  | 64.49 | 2826.6 |  | 64.49 | 310.8 |
| 64.54 | 1136.8 |  | 64.54 | 2727.2 |  | 64.54 | 309.4 |
| 64.59 | 1092 |  | 64.59 | 2713.2 |  | 64.59 | 280 |
| 64.64 | 1026.2 |  | 64.64 | 2660 |  | 64.64 | 277.2 |
| 64.69 | 1022 |  | 64.69 | 2662.8 |  | 64.69 | 291.2 |
| 64.74 | 1120 |  | 64.74 | 2577.4 |  | 64.74 | 309.4 |
| 64.79 | 1064 |  | 64.79 | 2704.8 |  | 64.79 | 282.8 |
| 64.84 | 1008 |  | 64.84 | 2660 |  | 64.84 | 282.8 |
| 64.89 | 1012.2 |  | 64.89 | 2661.4 |  | 64.89 | 282.8 |
| 64.94 | 992.6 |  | 64.94 | 2800 |  | 64.94 | 534.8 |
| 64.99 | 1040.2 |  | 64.99 | 2672.6 |  | 64.99 | 1050 |
| 65.04 | 977.2 |  | 65.04 | 2742.6 |  | 65.04 | 1054.2 |
| 65.09 | 981.4 |  | 65.09 | 2741.2 |  | 65.09 | 604.8 |
| 65.14 | 1009.4 |  | 65.14 | 2916.2 |  | 65.14 | 585.2 |
| 65.19 | 1052.8 |  | 65.19 | 2910.6 |  | 65.19 | 715.4 |
| 65.24 | 1120 |  | 65.24 | 2870 |  | 65.24 | 715.4 |
| 65.29 | 1122.8 |  | 65.29 | 2872.8 |  | 65.29 | 348.6 |
| 65.34 | 1177.4 |  | 65.34 | 2830.8 |  | 65.34 | 306.6 |
| 65.39 | 1178.8 |  | 65.39 | 2998.8 |  | 65.39 | 236.6 |
| 65.44 | 1374.8 |  | 65.44 | 3052 |  | 65.44 | 235.2 |
| 65.49 | 1374.8 |  | 65.49 | 3052 |  | 65.49 | 320.6 |
| 65.54 | 1107.4 |  | 65.54 | 2888.2 |  | 65.54 | 337.4 |
| 65.59 | 1132.6 |  | 65.59 | 3008.6 |  | 65.59 | 348.6 |
| 65.64 | 1163.4 |  | 65.64 | 2840.6 |  | 65.64 | 351.4 |
| 65.69 | 1160.6 |  | 65.69 | 2842 |  | 65.69 | 379.4 |
| 65.74 | 1052.8 |  | 65.74 | 2703.4 |  | 65.74 | 365.4 |
| 65.79 | 1110.2 |  | 65.79 | 2884 |  | 65.79 | 338.8 |
| 65.84 | 1012.2 |  | 65.84 | 2854.6 |  | 65.84 | 334.6 |
| 65.89 | 1006.6 |  | 65.89 | 2857.4 |  | 65.89 | 294 |
| 65.94 | 1008 |  | 65.94 | 2969.4 |  | 65.94 | 295.4 |
| 65.99 | 1026.2 |  | 65.99 | 2840.6 |  | 65.99 | 282.8 |
| 66.04 | 1080.8 |  | 66.04 | 2858.8 |  | 66.04 | 282.8 |
| 66.09 | 1080.8 |  | 66.09 | 2858.8 |  | 66.09 | 282.8 |
| 66.14 | 1009.4 |  | 66.14 | 2714.6 |  | 66.14 | 236.6 |
| 66.19 | 1075.2 |  | 66.19 | 2731.4 |  | 66.19 | 236.6 |
| 66.24 | 1024.8 |  | 66.24 | 2730 |  | 66.24 | 238 |
| 66.29 | 1024.8 |  | 66.29 | 2728.6 |  | 66.29 | 253.4 |
| 66.34 | 1104.6 |  | 66.34 | 2674 |  | 66.34 | 226.8 |
| 66.39 | 1038.8 |  | 66.39 | 2801.4 |  | 66.39 | 295.4 |
| 66.44 | 924 |  | 66.44 | 2755.2 |  | 66.44 | 292.6 |
| 66.49 | 926.8 |  | 66.49 | 2758 |  | 66.49 | 236.6 |
| 66.54 | 939.4 |  | 66.54 | 2647.4 |  | 66.54 | 249.2 |
| 66.59 | 924 |  | 66.59 | 2772 |  | 66.59 | 242.2 |
| 66.64 | 1010.8 |  | 66.64 | 2602.6 |  | 66.64 | 238 |
| 66.69 | 1006.6 |  | 66.69 | 2605.4 |  | 66.69 | 263.2 |
| 66.74 | 897.4 |  | 66.74 | 2801.4 |  | 66.74 | 240.8 |
| 66.79 | 984.2 |  | 66.79 | 2776.2 |  | 66.79 | 240.8 |
| 66.84 | 924 |  | 66.84 | 2800 |  | 66.84 | 242.2 |
| 66.89 | 922.6 |  | 66.89 | 2797.2 |  | 66.89 | 239.4 |
| 66.94 | 952 |  | 66.94 | 2914.8 |  | 66.94 | 266 |
| 66.99 | 953.4 |  | 66.99 | 2872.8 |  | 66.99 | 268.8 |
| 67.04 | 953.4 |  | 67.04 | 2910.6 |  | 67.04 | 266 |
| 67.09 | 953.4 |  | 67.09 | 2910.6 |  | 67.09 | 263.2 |
| 67.14 | 996.8 |  | 67.14 | 2888.2 |  | 67.14 | 250.6 |
| 67.19 | 982.8 |  | 67.19 | 2727.2 |  | 67.19 | 254.8 |
| 67.24 | 1068.2 |  | 67.24 | 2632 |  | 67.24 | 240.8 |
| 67.29 | 1065.4 |  | 67.29 | 2630.6 |  | 67.29 | 169.4 |
| 67.34 | 1122.8 |  | 67.34 | 2769.2 |  | 67.34 | 211.4 |
| 67.39 | 1092 |  | 67.39 | 2604 |  | 67.39 | 208.6 |
| 67.44 | 1188.6 |  | 67.44 | 2646 |  | 67.44 | 208.6 |
| 67.49 | 1194.2 |  | 67.49 | 2650.2 |  | 67.49 | 250.6 |
| 67.54 | 1204 |  | 67.54 | 2798.6 |  | 67.54 | 212.8 |
| 67.59 | 1272.6 |  | 67.59 | 2634.8 |  | 67.59 | 210 |
| 67.64 | 1272.6 |  | 67.64 | 2658.6 |  | 67.64 | 224 |
| 67.69 | 1271.2 |  | 67.69 | 2662.8 |  | 67.69 | 224 |
| 67.74 | 1374.8 |  | 67.74 | 2844.8 |  | 67.74 | 212.8 |
| 67.79 | 1512 |  | 67.79 | 2632 |  | 67.79 | 208.6 |
| 67.84 | 1442 |  | 67.84 | 2800 |  | 67.84 | 253.4 |
| 67.89 | 1440.6 |  | 67.89 | 2802.8 |  | 67.89 | 242.2 |
| 67.94 | 1486.8 |  | 67.94 | 2804.2 |  | 67.94 | 165.2 |
| 67.99 | 1173.2 |  | 67.99 | 2675.4 |  | 67.99 | 166.6 |
| 68.04 | 1120 |  | 68.04 | 2758 |  | 68.04 | 250.6 |
| 68.09 | 1121.4 |  | 68.09 | 2759.4 |  | 68.09 | 242.2 |
| 68.14 | 1076.6 |  | 68.14 | 2594.2 |  | 68.14 | 224 |
| 68.19 | 996.8 |  | 68.19 | 2604 |  | 68.19 | 225.4 |
| 68.24 | 992.6 |  | 68.24 | 2674 |  | 68.24 | 249.2 |
| 68.29 | 992.6 |  | 68.29 | 2676.8 |  | 68.29 | 225.4 |
| 68.34 | 940.8 |  | 68.34 | 2678.2 |  | 68.34 | 252 |
| 68.39 | 996.8 |  | 68.39 | 2734.2 |  | 68.39 | 254.8 |
| 68.44 | 896 |  | 68.44 | 2830.8 |  | 68.44 | 197.4 |
| 68.49 | 896 |  | 68.49 | 2828 |  | 68.49 | 212.8 |
| 68.54 | 872.2 |  | 68.54 | 2812.6 |  | 68.54 | 236.6 |
| 68.59 | 912.8 |  | 68.59 | 2602.6 |  | 68.59 | 238 |
| 68.64 | 852.6 |  | 68.64 | 2788.8 |  | 68.64 | 221.2 |
| 68.69 | 851.2 |  | 68.69 | 2787.4 |  | 68.69 | 222.6 |
| 68.74 | 1008 |  | 68.74 | 2958.2 |  | 68.74 | 268.8 |
| 68.79 | 940.8 |  | 68.79 | 2620.8 |  | 68.79 | 264.6 |
| 68.84 | 896 |  | 68.84 | 2629.2 |  | 68.84 | 238 |
| 68.89 | 893.2 |  | 68.89 | 2633.4 |  | 68.89 | 196 |
| 68.94 | 922.6 |  | 68.94 | 2868.6 |  | 68.94 | 256.2 |
| 68.99 | 1065.4 |  | 68.99 | 2634.8 |  | 68.99 | 252 |
| 69.04 | 841.4 |  | 69.04 | 2730 |  | 69.04 | 254.8 |
| 69.09 | 842.8 |  | 69.09 | 2732.8 |  | 69.09 | 208.6 |
| 69.14 | 869.4 |  | 69.14 | 2662.8 |  | 69.14 | 180.6 |
| 69.19 | 924 |  | 69.19 | 2622.2 |  | 69.19 | 180.6 |
| 69.24 | 826 |  | 69.24 | 2646 |  | 69.24 | 222.6 |
| 69.29 | 826 |  | 69.29 | 2646 |  | 69.29 | 236.6 |
| 69.34 | 912.8 |  | 69.34 | 2534 |  | 69.34 | 266 |
| 69.39 | 926.8 |  | 69.39 | 2661.4 |  | 69.39 | 266 |
| 69.44 | 1024.8 |  | 69.44 | 2688 |  | 69.44 | 267.4 |
| 69.49 | 1023.4 |  | 69.49 | 2688 |  | 69.49 | 236.6 |
| 69.54 | 938 |  | 69.54 | 2662.8 |  | 69.54 | 256.2 |
| 69.59 | 914.2 |  | 69.59 | 2608.2 |  | 69.59 | 256.2 |
| 69.64 | 949.2 |  | 69.64 | 2728.6 |  | 69.64 | 264.6 |
| 69.69 | 950.6 |  | 69.69 | 2730 |  | 69.69 | 165.2 |
| 69.74 | 1093.4 |  | 69.74 | 2548 |  | 69.74 | 211.4 |
| 69.79 | 1201.2 |  | 69.79 | 2606.8 |  | 69.79 | 212.8 |
| 69.84 | 1134 |  | 69.84 | 2718.8 |  | 69.84 | 238 |
| 69.89 | 1134 |  | 69.89 | 2714.6 |  | 69.89 | 236.6 |
| 69.94 | 1248.8 |  | 69.94 | 2770.6 |  | 69.94 | 224 |
| 69.99 | 1442 |  | 69.99 | 2730 |  | 69.99 | 221.2 |
| 70.04 | 1460.2 |  | 70.04 | 2914.8 |  | 70.04 | 240.8 |
| 70.09 | 1454.6 |  | 70.09 | 2909.2 |  | 70.09 | 252 |
| 70.14 | 1401.4 |  | 70.14 | 2997.4 |  | 70.14 | 211.4 |
| 70.19 | 1360.8 |  | 70.19 | 2955.4 |  | 70.19 | 211.4 |
| 70.24 | 1202.6 |  | 70.24 | 2886.8 |  | 70.24 | 210 |
| 70.29 | 1205.4 |  | 70.29 | 2884 |  | 70.29 | 254.8 |
| 70.34 | 1145.2 |  | 70.34 | 2956.8 |  | 70.34 | 256.2 |
| 70.39 | 1122.8 |  | 70.39 | 2912 |  | 70.39 | 250.6 |
| 70.44 | 1135.4 |  | 70.44 | 2868.6 |  | 70.44 | 212.8 |
| 70.49 | 1136.8 |  | 70.49 | 2872.8 |  | 70.49 | 322 |
| 70.54 | 1080.8 |  | 70.54 | 2930.2 |  | 70.54 | 208.6 |
| 70.59 | 1106 |  | 70.59 | 2942.8 |  | 70.59 | 210 |
| 70.64 | 1107.4 |  | 70.64 | 2748.2 |  | 70.64 | 238 |
| 70.69 | 1107.4 |  | 70.69 | 2744 |  | 70.69 | 225.4 |
| 70.74 | 1192.8 |  | 70.74 | 2857.4 |  | 70.74 | 239.4 |
| 70.79 | 980 |  | 70.79 | 2832.2 |  | 70.79 | 235.2 |
| 70.84 | 1012.2 |  | 70.84 | 2802.8 |  | 70.84 | 222.6 |
| 70.89 | 1008 |  | 70.89 | 2802.8 |  | 70.89 | 208.6 |
| 70.94 | 907.2 |  | 70.94 | 2888.2 |  | 70.94 | 198.8 |
| 70.99 | 908.6 |  | 70.99 | 2755.2 |  | 70.99 | 194.6 |
| 71.04 | 928.2 |  | 71.04 | 2676.8 |  | 71.04 | 253.4 |
| 71.09 | 924 |  | 71.09 | 2676.8 |  | 71.09 | 222.6 |
| 71.14 | 954.8 |  | 71.14 | 2938.6 |  | 71.14 | 228.2 |
| 71.19 | 908.6 |  | 71.19 | 2815.4 |  | 71.19 | 226.8 |
| 71.24 | 882 |  | 71.24 | 2686.6 |  | 71.24 | 253.4 |
| 71.29 | 880.6 |  | 71.29 | 2690.8 |  | 71.29 | 193.2 |
| 71.34 | 858.2 |  | 71.34 | 2870 |  | 71.34 | 253.4 |
| 71.39 | 1047.2 |  | 71.39 | 2706.2 |  | 71.39 | 252 |
| 71.44 | 970.2 |  | 71.44 | 2706.2 |  | 71.44 | 212.8 |
| 71.49 | 967.4 |  | 71.49 | 2857.4 |  | 71.49 | 210 |
| 71.54 | 952 |  | 71.54 | 2742.6 |  | 71.54 | 208.6 |
| 71.59 | 967.4 |  | 71.59 | 2650.2 |  | 71.59 | 208.6 |
| 71.64 | 950.6 |  | 71.64 | 2647.4 |  | 71.64 | 225.4 |
| 71.69 | 952 |  | 71.69 | 2802.8 |  | 71.69 | 214.2 |
| 71.74 | 868 |  | 71.74 | 2816.8 |  | 71.74 | 169.4 |
| 71.79 | 896 |  | 71.79 | 2926 |  | 71.79 | 166.6 |
| 71.84 | 1020.6 |  | 71.84 | 2926 |  | 71.84 | 240.8 |
| 71.89 | 1022 |  | 71.89 | 2900.8 |  | 71.89 | 198.8 |
| 71.94 | 1082.2 |  | 71.94 | 2966.6 |  | 71.94 | 252 |
| 71.99 | 914.2 |  | 71.99 | 3236.8 |  | 71.99 | 249.2 |
| 72.04 | 952 |  | 72.04 | 3236.8 |  | 72.04 | 256.2 |
| 72.09 | 949.2 |  | 72.09 | 2968 |  | 72.09 | 240.8 |
| 72.14 | 978.6 |  | 72.14 | 2942.8 |  | 72.14 | 252 |
| 72.19 | 1104.6 |  | 72.19 | 2868.6 |  | 72.19 | 250.6 |
| 72.24 | 992.6 |  | 72.24 | 2867.2 |  | 72.24 | 256.2 |
| 72.29 | 996.8 |  | 72.29 | 2872.8 |  | 72.29 | 226.8 |
| 72.34 | 991.2 |  | 72.34 | 2788.8 |  | 72.34 | 242.2 |
| 72.39 | 968.8 |  | 72.39 | 3010 |  | 72.39 | 236.6 |
| 72.44 | 968.8 |  | 72.44 | 3007.2 |  | 72.44 | 239.4 |
| 72.49 | 925.4 |  | 72.49 | 2790.2 |  | 72.49 | 266 |
| 72.54 | 884.8 |  | 72.54 | 2774.8 |  | 72.54 | 222.6 |
| 72.59 | 827.4 |  | 72.59 | 2868.6 |  | 72.59 | 221.2 |
| 72.64 | 826 |  | 72.64 | 2872.8 |  | 72.64 | 196 |
| 72.69 | 810.6 |  | 72.69 | 3008.6 |  | 72.69 | 212.8 |
| 72.74 | 936.6 |  | 72.74 | 2884 |  | 72.74 | 211.4 |
| 72.79 | 953.4 |  | 72.79 | 2686.6 |  | 72.79 | 210 |
| 72.84 | 954.8 |  | 72.84 | 2686.6 |  | 72.84 | 222.6 |
| 72.89 | 910 |  | 72.89 | 2634.8 |  | 72.89 | 212.8 |
| 72.94 | 1121.4 |  | 72.94 | 2790.2 |  | 72.94 | 224 |
| 72.99 | 1092 |  | 72.99 | 2685.2 |  | 72.99 | 226.8 |
| 73.04 | 1093.4 |  | 73.04 | 2690.8 |  | 73.04 | 210 |
| 73.09 | 1068.2 |  | 73.09 | 2578.8 |  | 73.09 | 194.6 |
| 73.14 | 1066.8 |  | 73.14 | 2857.4 |  | 73.14 | 268.8 |
| 73.19 | 1048.6 |  | 73.19 | 2825.2 |  | 73.19 | 267.4 |
| 73.24 | 1052.8 |  | 73.24 | 2829.4 |  | 73.24 | 180.6 |
| 73.29 | 1220.8 |  | 73.29 | 2577.4 |  | 73.29 | 224 |
| 73.34 | 1122.8 |  | 73.34 | 2560.6 |  | 73.34 | 200.2 |
| 73.39 | 1194.2 |  | 73.39 | 2576 |  | 73.39 | 194.6 |
| 73.44 | 1194.2 |  | 73.44 | 2577.4 |  | 73.44 | 210 |
| 73.49 | 1012.2 |  | 73.49 | 2731.4 |  | 73.49 | 224 |
| 73.54 | 964.6 |  | 73.54 | 2661.4 |  | 73.54 | 240.8 |
| 73.59 | 1065.4 |  | 73.59 | 2713.2 |  | 73.59 | 242.2 |
| 73.64 | 1064 |  | 73.64 | 2717.4 |  | 73.64 | 235.2 |
| 73.69 | 940.8 |  | 73.69 | 2657.2 |  | 73.69 | 156.8 |
| 73.74 | 925.4 |  | 73.74 | 2620.8 |  | 73.74 | 211.4 |
| 73.79 | 882 |  | 73.79 | 2730 |  | 73.79 | 207.2 |
| 73.84 | 880.6 |  | 73.84 | 2732.8 |  | 73.84 | 266 |
| 73.89 | 907.2 |  | 73.89 | 2801.4 |  | 73.89 | 238 |
| 73.94 | 856.8 |  | 73.94 | 2644.6 |  | 73.94 | 226.8 |
| 73.99 | 992.6 |  | 73.99 | 2616.6 |  | 73.99 | 226.8 |
| 74.04 | 991.2 |  | 74.04 | 2619.4 |  | 74.04 | 252 |
| 74.09 | 1050 |  | 74.09 | 2605.4 |  | 74.09 | 228.2 |
| 74.14 | 1148 |  | 74.14 | 2578.8 |  | 74.14 | 249.2 |
| 74.19 | 1313.2 |  | 74.19 | 2787.4 |  | 74.19 | 252 |
| 74.24 | 1318.8 |  | 74.24 | 2786 |  | 74.24 | 235.2 |
| 74.29 | 1216.6 |  | 74.29 | 2563.4 |  | 74.29 | 221.2 |
| 74.34 | 1327.2 |  | 74.34 | 2689.4 |  | 74.34 | 222.6 |
| 74.39 | 1178.8 |  | 74.39 | 2577.4 |  | 74.39 | 221.2 |
| 74.44 | 1178.8 |  | 74.44 | 2576 |  | 74.44 | 184.8 |
| 74.49 | 1206.8 |  | 74.49 | 2604 |  | 74.49 | 172.2 |
| 74.54 | 1089.2 |  | 74.54 | 2564.8 |  | 74.54 | 169.4 |
| 74.59 | 1076.096 |  | 74.59 | 2893.139 |  | 74.59 | 172.3704 |
| 74.64 | 1074.92 |  | 74.64 | 2892.391 |  | 74.64 | 171.8698 |
| 74.69 | 1073.744 |  | 74.69 | 2891.642 |  | 74.69 | 171.3693 |
| 74.74 | 1072.569 |  | 74.74 | 2890.894 |  | 74.74 | 170.8688 |
| 74.79 | 1071.393 |  | 74.79 | 2890.146 |  | 74.79 | 170.3683 |
| 74.84 | 1070.217 |  | 74.84 | 2889.398 |  | 74.84 | 169.8678 |
| 74.89 | 1069.041 |  | 74.89 | 2888.65 |  | 74.89 | 169.3673 |
| 74.94 | 1067.866 |  | 74.94 | 2887.902 |  | 74.94 | 168.8667 |
| 74.99 | 1066.69 |  | 74.99 | 2887.154 |  | 74.99 | 168.3662 |
| 75.04 | 1065.514 |  | 75.04 | 2886.405 |  | 75.04 | 167.8657 |
| 75.09 | 1064.338 |  | 75.09 | 2885.657 |  | 75.09 | 167.3652 |
| 75.14 | 1063.163 |  | 75.14 | 2884.909 |  | 75.14 | 166.8647 |
| 75.19 | 1061.987 |  | 75.19 | 2884.161 |  | 75.19 | 166.3642 |
| 75.24 | 1060.811 |  | 75.24 | 2883.413 |  | 75.24 | 165.8636 |
| 75.29 | 1059.635 |  | 75.29 | 2882.665 |  | 75.29 | 165.3631 |
| 75.34 | 1058.46 |  | 75.34 | 2881.917 |  | 75.34 | 164.8626 |
| 75.39 | 1057.284 |  | 75.39 | 2881.168 |  | 75.39 | 164.3621 |
| 75.44 | 1056.108 |  | 75.44 | 2880.42 |  | 75.44 | 163.8616 |
| 75.49 | 1054.932 |  | 75.49 | 2879.672 |  | 75.49 | 163.361 |
| 75.54 | 1053.757 |  | 75.54 | 2878.924 |  | 75.54 | 162.8605 |
| 75.59 | 1052.581 |  | 75.59 | 2878.176 |  | 75.59 | 162.36 |
| 75.64 | 1051.405 |  | 75.64 | 2877.428 |  | 75.64 | 161.8595 |
| 75.69 | 1050.229 |  | 75.69 | 2876.68 |  | 75.69 | 161.359 |
| 75.74 | 1049.054 |  | 75.74 | 2875.931 |  | 75.74 | 160.8585 |
| 75.79 | 1047.878 |  | 75.79 | 2875.183 |  | 75.79 | 160.3579 |
| 75.84 | 1046.702 |  | 75.84 | 2874.435 |  | 75.84 | 159.8574 |
| 75.89 | 1045.526 |  | 75.89 | 2873.687 |  | 75.89 | 159.3569 |
| 75.94 | 1044.351 |  | 75.94 | 2872.939 |  | 75.94 | 158.8564 |
| 75.99 | 1043.175 |  | 75.99 | 2872.191 |  | 75.99 | 158.3559 |
| 76.04 | 1041.999 |  | 76.04 | 2871.442 |  | 76.04 | 157.8553 |
| 76.09 | 1040.823 |  | 76.09 | 2870.694 |  | 76.09 | 157.3548 |
| 76.14 | 1039.648 |  | 76.14 | 2869.946 |  | 76.14 | 156.8543 |
| 76.19 | 1038.472 |  | 76.19 | 2869.198 |  | 76.19 | 156.3538 |
| 76.24 | 1037.296 |  | 76.24 | 2868.45 |  | 76.24 | 155.8533 |
| 76.29 | 1036.12 |  | 76.29 | 2867.702 |  | 76.29 | 155.3528 |
| 76.34 | 1034.945 |  | 76.34 | 2866.954 |  | 76.34 | 154.8522 |
| 76.39 | 1033.769 |  | 76.39 | 2866.205 |  | 76.39 | 154.3517 |
| 76.44 | 1032.593 |  | 76.44 | 2865.457 |  | 76.44 | 153.8512 |
| 76.49 | 1031.417 |  | 76.49 | 2864.709 |  | 76.49 | 153.3507 |
| 76.54 | 1030.242 |  | 76.54 | 2863.961 |  | 76.54 | 152.8502 |
| 76.59 | 1029.066 |  | 76.59 | 2863.213 |  | 76.59 | 152.3497 |
| 76.64 | 1027.89 |  | 76.64 | 2862.465 |  | 76.64 | 151.8491 |
| 76.69 | 1026.714 |  | 76.69 | 2861.717 |  | 76.69 | 151.3486 |
| 76.74 | 1025.539 |  | 76.74 | 2860.968 |  | 76.74 | 150.8481 |
| 76.79 | 1024.363 |  | 76.79 | 2860.22 |  | 76.79 | 150.3476 |
| 76.84 | 1023.187 |  | 76.84 | 2859.472 |  | 76.84 | 149.8471 |
| 76.89 | 1022.011 |  | 76.89 | 2858.724 |  | 76.89 | 149.3465 |
| 76.94 | 1020.836 |  | 76.94 | 2857.976 |  | 76.94 | 148.846 |
| 76.99 | 1019.66 |  | 76.99 | 2857.228 |  | 76.99 | 148.3455 |
| 77.04 | 1018.484 |  | 77.04 | 2856.479 |  | 77.04 | 147.845 |
| 77.09 | 1017.308 |  | 77.09 | 2855.731 |  | 77.09 | 147.3445 |
| 77.14 | 1016.133 |  | 77.14 | 2854.983 |  | 77.14 | 146.844 |
| 77.19 | 1014.957 |  | 77.19 | 2854.235 |  | 77.19 | 146.3434 |
| 77.24 | 1013.781 |  | 77.24 | 2853.487 |  | 77.24 | 145.8429 |
| 77.29 | 1012.605 |  | 77.29 | 2852.739 |  | 77.29 | 145.3424 |
| 77.34 | 1011.43 |  | 77.34 | 2851.991 |  | 77.34 | 144.8419 |
| 77.39 | 1010.254 |  | 77.39 | 2851.242 |  | 77.39 | 144.3414 |
| 77.44 | 1009.078 |  | 77.44 | 2850.494 |  | 77.44 | 143.8409 |
| 77.49 | 1007.902 |  | 77.49 | 2849.746 |  | 77.49 | 143.3403 |
| 77.54 | 1006.727 |  | 77.54 | 2848.998 |  | 77.54 | 142.8398 |
| 77.59 | 1005.551 |  | 77.59 | 2848.25 |  | 77.59 | 142.3393 |
| 77.64 | 1004.375 |  | 77.64 | 2847.502 |  | 77.64 | 141.8388 |
| 77.69 | 1003.2 |  | 77.69 | 2846.754 |  | 77.69 | 141.3383 |
| 77.74 | 1002.024 |  | 77.74 | 2846.005 |  | 77.74 | 140.8377 |
| 77.79 | 1000.848 |  | 77.79 | 2845.257 |  | 77.79 | 140.3372 |
| 77.84 | 999.6723 |  | 77.84 | 2844.509 |  | 77.84 | 139.8367 |
| 77.89 | 998.4965 |  | 77.89 | 2843.761 |  | 77.89 | 139.3362 |
| 77.94 | 997.3208 |  | 77.94 | 2843.013 |  | 77.94 | 138.8357 |
| 77.99 | 996.145 |  | 77.99 | 2842.265 |  | 77.99 | 138.3352 |
| 78.04 | 994.9693 |  | 78.04 | 2841.517 |  | 78.04 | 137.8346 |
| 78.09 | 993.7935 |  | 78.09 | 2840.768 |  | 78.09 | 137.3341 |
| 78.14 | 992.6178 |  | 78.14 | 2840.02 |  | 78.14 | 136.8336 |
| 78.19 | 991.442 |  | 78.19 | 2839.272 |  | 78.19 | 136.3331 |
| 78.24 | 990.2663 |  | 78.24 | 2838.524 |  | 78.24 | 135.8326 |
| 78.29 | 989.0905 |  | 78.29 | 2837.776 |  | 78.29 | 135.3321 |
| 78.34 | 987.9148 |  | 78.34 | 2837.028 |  | 78.34 | 134.8315 |
| 78.39 | 986.739 |  | 78.39 | 2836.279 |  | 78.39 | 134.331 |
| 78.44 | 985.5633 |  | 78.44 | 2835.531 |  | 78.44 | 133.8305 |
| 78.49 | 984.3876 |  | 78.49 | 2834.783 |  | 78.49 | 133.33 |
| 78.54 | 983.2118 |  | 78.54 | 2834.035 |  | 78.54 | 132.8295 |
| 78.59 | 982.0361 |  | 78.59 | 2833.287 |  | 78.59 | 132.3289 |
| 78.64 | 980.8603 |  | 78.64 | 2832.539 |  | 78.64 | 131.8284 |
| 78.69 | 979.6846 |  | 78.69 | 2831.791 |  | 78.69 | 131.3279 |
| 78.74 | 978.5088 |  | 78.74 | 2831.042 |  | 78.74 | 130.8274 |
| 78.79 | 977.3331 |  | 78.79 | 2830.294 |  | 78.79 | 130.3269 |
| 78.84 | 976.1573 |  | 78.84 | 2829.546 |  | 78.84 | 129.8264 |
| 78.89 | 974.9816 |  | 78.89 | 2828.798 |  | 78.89 | 129.3258 |
| 78.94 | 973.8058 |  | 78.94 | 2828.05 |  | 78.94 | 128.8253 |
| 78.99 | 972.6301 |  | 78.99 | 2827.302 |  | 78.99 | 128.3248 |
| 79.04 | 971.4543 |  | 79.04 | 2826.554 |  | 79.04 | 127.8243 |
| 79.09 | 970.2786 |  | 79.09 | 2825.805 |  | 79.09 | 127.3238 |
| 79.14 | 969.1028 |  | 79.14 | 2825.057 |  | 79.14 | 126.8232 |
| 79.19 | 967.9271 |  | 79.19 | 2824.309 |  | 79.19 | 126.3227 |
| 79.24 | 966.7513 |  | 79.24 | 2823.561 |  | 79.24 | 125.8222 |
| 79.29 | 965.5756 |  | 79.29 | 2822.813 |  | 79.29 | 125.3217 |
| 79.34 | 964.3999 |  | 79.34 | 2822.065 |  | 79.34 | 124.8212 |
| 79.39 | 963.2241 |  | 79.39 | 2821.317 |  | 79.39 | 124.3207 |
| 79.44 | 962.0484 |  | 79.44 | 2820.568 |  | 79.44 | 123.8201 |
| 79.49 | 960.8726 |  | 79.49 | 2819.82 |  | 79.49 | 123.3196 |
| 79.54 | 959.6969 |  | 79.54 | 2819.072 |  | 79.54 | 122.8191 |
| 79.59 | 958.5211 |  | 79.59 | 2818.324 |  | 79.59 | 122.3186 |
| 79.64 | 957.3454 |  | 79.64 | 2817.576 |  | 79.64 | 121.8181 |
| 79.69 | 956.1696 |  | 79.69 | 2816.828 |  | 79.69 | 121.3176 |
| 79.74 | 954.9939 |  | 79.74 | 2816.079 |  | 79.74 | 120.817 |
| 79.79 | 953.8181 |  | 79.79 | 2815.331 |  | 79.79 | 120.3165 |
| 79.84 | 952.6424 |  | 79.84 | 2814.583 |  | 79.84 | 119.816 |
| 79.89 | 951.4666 |  | 79.89 | 2813.835 |  | 79.89 | 119.3155 |
| 79.94 | 950.2909 |  | 79.94 | 2813.087 |  | 79.94 | 118.815 |
| 79.99 | 949.1151 |  | 79.99 | 2812.339 |  | 79.99 | 118.3144 |
