# Supplementary material for: Application of synthesized metal-trimesic acid frameworks for the remediation of a multi-metal polluted soil and investigation of quinoa responses
Source: PLoS One. 2024 Sep 6;19(9):e0310054. doi: 10.1371/journal.pone.0310054 (PMC11379216; doi:10.1371/journal.pone.0310054)
Supplement: Attachment — Submitted filename: Response to Reviewers.docx [file pone.0310054.s005.docx]

**Dear professor Zhongchuang Liu**

**Academic Editor of PLOS ONE**

Thank you very much for considering our article.

The revisions have been done according to what you and reviewers asked.

*******

Dear professor Zhongchuang Liu

1. The manuscript has met PLOS ONE's style requirements, including those for file naming and the PLOS ONE style templates.
2. The captions for Supporting Information files were included at the end of our manuscript according to Supporting Information guidelines.

Your suggested articles have been used in the present article in the sections of Introduction and Materials and methods and they have been marked with **green** font color in the text.

Dear Reviewer #1

- The quality of the images has been adjusted by PLOS ONE Pace Image Tool. However, the length and width of the images have been increased again as much as possible.
- The scientific names of plants have been written in italics and marked in **light orange** font color in the text.

Dear Reviewer #2

Your recommended revisions have been marked with **purple** font color in the text:

- The study's objectives at the beginning, the significance and implications of the findings for environmental remediation practices have been more clearly stated in the Abstract section.
- The reason for choosing quinoa for this study is given in the Introduction section. Old references have been replaced with new references (2024) in this section.
- Statistical analysis is described in more detail in the Materials and Methods section.
- The limitations and problems of conducting the experiment are explained under the heading of Potential limitations of the study and suggestions in the Results and discussion section. The significant role of copper in the production of high-performance MOFs is also mentioned. The use of copper MOFs in acidic contaminated soils has been mentioned. Also, the use of other organic ligands to synthesis MOFs is suggested for future studies.
- In the conclusion section, the high efficiency of copper MOF is mentioned due to its unique structure, especially for the removal of Pb in polluted urban soils.
- A number of new references (2024) have been replaced with old references or added in References section.

Dear Reviewer #3

Your recommended revisions have been marked with **light blue** font color in the text:

- The previous title of the article has been changed according to your recommendation.
- "belongs to" has been replaced by other appropriate words throughout the text.
- Heavy metal absorption by quinoa in this study has been compared to other studies.
- Many grammatical corrections have been made in the text, marked by Track Changes.

Best regards

Dr. Reza Ghasemi-Fasaei

Corresponding author
