# Supplementary material for: Application of synthesized metal-trimesic acid frameworks for the remediation of a multi-metal polluted soil and investigation of quinoa responses
Source: PLoS One. 2024 Sep 6;19(9):e0310054. doi: 10.1371/journal.pone.0310054 (PMC11379216; doi:10.1371/journal.pone.0310054)
Supplement: Attachment — Submitted filename: Response to Reviewers.docx [file pone.0310054.s006.docx]

**Dear professor Zhongchuang Liu**

**Academic Editor of PLOS ONE**

Thank you very much again for considering our article.

The second round of revisions have been done according to what you recommended.

*******

Dear professor Zhongchuang Liu

- There was a retracted reference that was replaced by another reference. In reference number 35, Haseeb et al. 2022 was replaced by Bamagoos et al. 2022.
- The scientific names of plants that were not written in Italic in the references section were corrected.
- Fig 1 was edited and the name of the vertical axis is now clear.

Best regards

Dr. Reza Ghasemi-Fasaei

Corresponding author
